# Vegetable omega-3 and omega-6 fatty acids differentially modulate the antiviral and antibacterial immune responses of Atlantic salmon

**DOI:** 10.1038/s41598-024-61144-w

**Published:** 2024-05-13

**Authors:** Albert Caballero-Solares, Khalil Eslamloo, Jennifer R. Hall, Tomer Katan, Mohamed Emam, Xi Xue, Richard G. Taylor, Rachel Balder, Christopher C. Parrish, Matthew L. Rise

**Affiliations:** 1 Department of Ocean Sciences, Memorial University of Newfoundland, St. John’s, NL Canada; 2 Aquatic Research Cluster, CREAIT Network, Memorial University of Newfoundland, St. John’s, NL Canada; 3 Cargill Animal Nutrition and Health, Elk River, MN USA; 4 Present Address: Centre for Marine Applied Research, Dartmouth, NS Canada; 5 Present Address: Stantec Inc., St. John’s, NL Canada

**Keywords:** Immunology, Molecular biology

## Abstract

The immunomodulatory effects of omega-3 and omega-6 fatty acids are a crucial subject of investigation for sustainable fish aquaculture, as fish oil is increasingly replaced by terrestrial vegetable oils in aquafeeds. Unlike previous research focusing on fish oil replacement with vegetable alternatives, our study explored how the omega-6 to omega-3 polyunsaturated fatty acid (PUFA) ratio in low-fish oil aquafeeds influences Atlantic salmon's antiviral and antibacterial immune responses. Atlantic salmon were fed aquafeeds rich in soy oil (high in omega-6) or linseed oil (high in omega-3) for 12 weeks and then challenged with bacterial (formalin-killed *Aeromonas salmonicida*) or viral-like (polyriboinosinic polyribocytidylic acid) antigens. The head kidneys of salmon fed high dietary omega-3 levels exhibited a more anti-inflammatory fatty acid profile and a restrained induction of pro-inflammatory and neutrophil-related genes during the immune challenges. The high-omega-3 diet also promoted a higher expression of genes associated with the interferon-mediated signaling pathway, potentially enhancing antiviral immunity. This research highlights the capacity of vegetable oils with different omega-6 to omega-3 PUFA ratios to modulate specific components of fish immune responses, offering insights for future research on the intricate lipid nutrition-immunity interplay and the development of novel sustainable low-fish oil clinical aquaculture feeds.

## Introduction

The immunomodulatory properties of omega-3 (ω3) and omega-6 (ω6) fatty acids (FAs) have been studied from a human health perspective since the 1970s^1^. For fish, this area of research emerged in the early 1990s^2^ and gained importance with the progressive replacement of wild-fish-derived marine ingredients [e.g., fish oil (FO)] in aquafeeds for farmed fish with more sustainable alternatives of terrestrial origin. Owing to their wide availability and competitive price, terrestrial plants have become a fundamental source of nutrients [e.g., vegetable oils (VOs)] for global aquaculture^3^.

FAs play essential structural and functional roles in the immune system of all living organisms^4^. As components of cell membranes, they define their physicochemical properties (e.g., fluidity) and ability to reconfigure in response to immune stimuli^4^. Moreover, long-chain polyunsaturated FAs (LC-PUFAs) such as eicosapentaenoic acid (20:5ω3; EPA), docosahexaenoic acid (22:6ω3; DHA), and arachidonic acid (20:4ω6; ARA) are precursors of anti-inflammatory (EPA, DHA-derived) and pro-inflammatory (ARA-derived) eicosanoids^1,4,5^. EPA’s and DHA’s health-promoting properties in humans are well-known^1,5^ –therefore, EPA + DHA-rich products (e.g., marine fish) are perceived as healthy foods by human consumers.

Except for some transgenic lines, VOs are devoid of LC-PUFAs, which are naturally abundant in wild marine fishes –and in FO, consequently^6^. Although current low-FO/high-VO aquafeed formulations effectively fuel fish growth, they inevitably lead to lower tissue EPA and DHA proportions^7^. Besides its potential impact on aquaculture product marketability, these constitutive lipid composition changes alter fish immunity and disease resistance^4,8–16^.

In reaction to the industry’s challenge of sustainable fish nutrition, the investigation of dietary FA effects on fish immune responses has focused on FO-replacement/VO-inclusion in aquafeeds. The effects of increasing VO levels in aquafeeds range from immunosuppressive^8–10,12,13^ to immunostimulatory^11,14,15^. The type of VO used for FO replacement is likely a major contributor to the opposing immunomodulatory effects. VOs can be high in ω6 polyunsaturated FAs [ω6 PUFAs; namely, linoleic acid (18:2ω6; LNA)], ω3 PUFAs [namely, α-linolenic acid (18:3ω3; ALA)], monounsaturated FAs (MUFAs), and saturated FAs (SFAs)^6^. The selection of VOs for inclusion in the diet will determine immune-relevant dietary lipid parameters, such as the balance between ω3 and ω6 FAs in the diet^4,16–20^.

Few studies have addressed the physiological effects of the dietary ω6:ω3 ratio without modifying the FO inclusion level in the aquafeeds (i.e., its EPA + DHA levels). Atlantic salmon fed a low-FO aquafeed with high inclusion of linseed oil (ALA-rich; with a ω6:ω3 ratio of 0.4) increased the hepatic transcript levels of genes involved in the crosstalk between lipid homeostasis and immunity [e.g., *oxysterols receptor LXR-alpha* (*lxra*)] compared with salmon fed a low-FO high-soy oil aquafeed (LNA-rich; with a ω6:ω3 ratio of 2.7)^18,19,21,22^. Emam et al.^16^ showed that switching between a high-LNA to a high-ALA diet modulated Atlantic salmon’s head kidney immune gene expression response to formalin-killed *Renibacterium salmoninarum*. Therefore, in addition to being necessary for sustainability, dietary FO replacement with VOs may be an opportunity to develop novel clinical aquafeeds for fish aquaculture.

The present study investigated how very different ω6:ω3 ratios in low-FO aquafeeds affect the Atlantic salmon head kidney antiviral and antibacterial immune responses via quantitative real-time polymerase chain reaction (RT-qPCR). The mRNA levels of 46 immune-relevant genes were analyzed in the head kidneys of Atlantic salmon post-smolts fed either a high-soy oil diet (High-ω6) or a high-linseed oil diet (High-ω3) for 12 weeks and intraperitoneally injected with either phosphate-buffered saline (PBS), formalin-killed *Aeromonas salmonicida* (Asal), or polyriboinosinic polyribocytidylic acid [poly(I:C)]. To compare the diets’ gene expression modulatory effects to tissue lipid composition changes, we also analyzed the lipid class (e.g., phospholipids, sterols) and FA profiles of the head kidneys of non-challenged Atlantic salmon.

## Results

### Head kidney immune gene expression response to Asal and poly(I:C)

**Figure 1:**
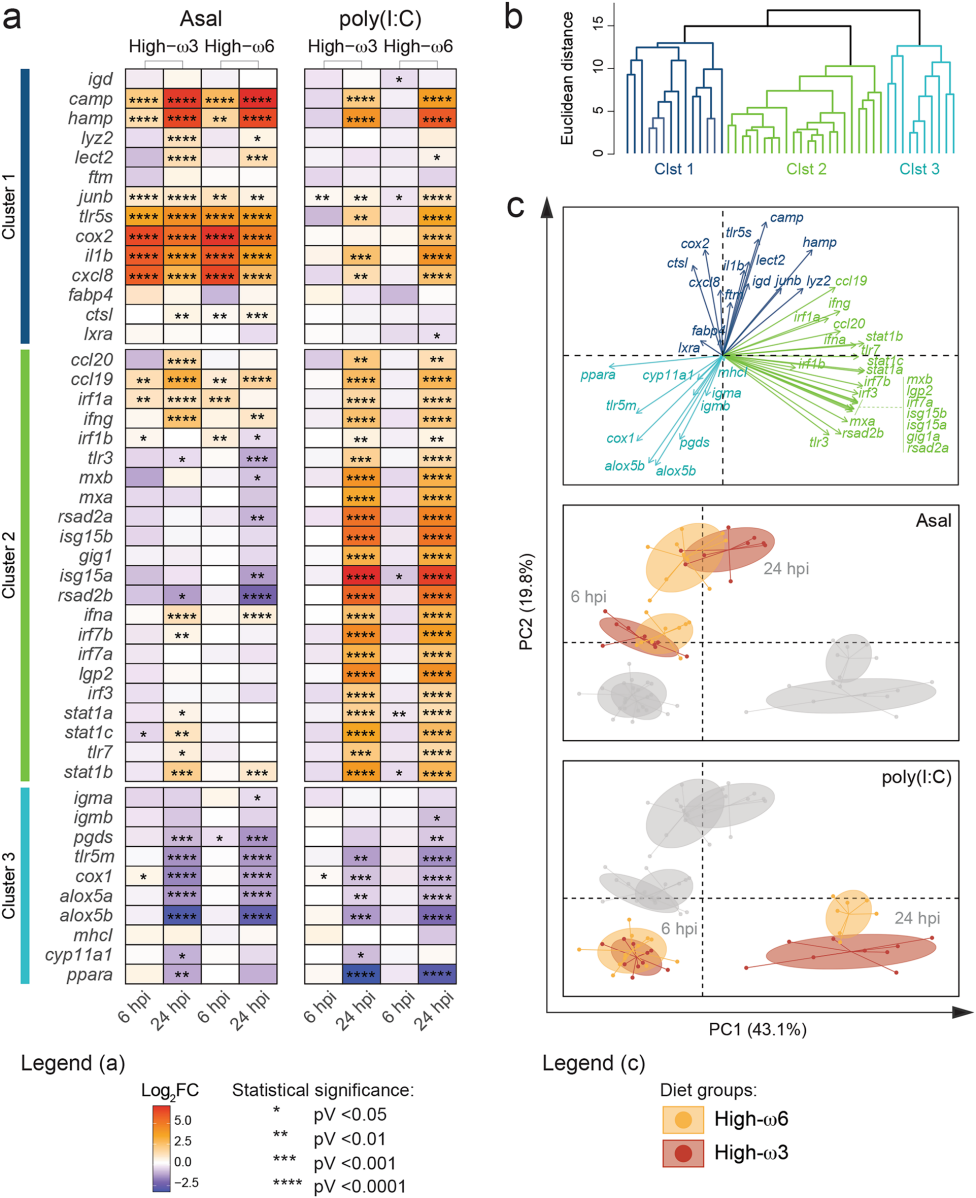
RT-qPCR analysis of the response of 46 immune-relevant genes to formalin-killed *A. salmonicida* (Asal) and polyriboinosinic polyribocytidylic acid [poly(I:C)]. (**a**) Heatmap representing the mean log_2_ fold-change of a given gene for each treatment [Asal and poly(I:C)], diet (High-ω3 and High-ω6), and time point (6 and 24 hpi) combination. Asterisks indicate significant transcript abundance differences between the Asal/poly(I:C)-injected salmon and the PBS-injected (n = 7–12 in each group). Data normal distribution and homoscedasticity were assessed using the Shapiro–Wilk and Levene’s tests, respectively. Contrast analysis was performed via Student’s t-test (if parametric) or Mann–Whitney U test (if non-parametric or heteroscedastic). An alpha value of 0.05 was applied to all tests. (**b**) Complete linkage hierarchical clustering analysis. (**c**) Principal Component Analysis (PCA) plots showing the loading vectors (top), the distribution of the Asal-injected salmon (middle), and the distribution of the poly(I:C)-injected salmon (bottom). The genes’ loading vectors are colored based on the hierarchical clustering, whereas the dots representing the salmon individuals are colored by diet group. All salmon individual dots are connected to the group centroid, which is surrounded by an ellipse representing their 95% confidence interval.

In this study, the hierarchical analysis of the log_2_-transformed fold-changes (log_2_FCs) grouped the genes of interest into three major clusters (Fig. 1a,b) with distinctive expression patterns.

Asal significantly induced most genes in Cluster 1 [i.e., significantly higher transcript levels than in the diet- and time-matched PBS-injected salmon; *p*-value (pV) < 0.05] (Fig. 1a; Supplementary Data 1; Supplementary Fig. 1). Regardless of diet, *cathelicidin antimicrobial peptide* (*camp*), *hepcidin* (*hamp*), *transcription factor JunB* (*junb*), *toll-like receptor 5, secreted* (*tlr5s*), *cyclooxygenase-2* (*cox2*), *interleukin-1 beta* (*il1b*), *interleukin-8* (*cxcl8*) were significantly induced at both 6 and 24 hpi, and *lysozyme C II* (*lyz2*) and *leukocyte cell-derived chemotaxin-2* (*lect2*) only at 24 hpi. As for *cathepsin L1* (*ctsl*), its mRNA levels were significantly induced at both time points in the High-ω6-fed salmon, but only at 24 hpi in the High-ω3-fed.

At 24 hpi, some Cluster 1 genes were significantly poly(I:C)-induced (i.e., *camp*, *hamp*, *junb*, *tlr5s*, *il1b*, and *cxcl8*) in both diet groups, whereas others (i.e., *lect2*, and *cox2*) were only poly(I:C)-induced in the High-ω6-fed salmon. Notably, *junb* was significantly poly(I:C)-induced at 6 hpi in the High-ω3-fed salmon but repressed at 6 hpi in the High-ω6-fed salmon. High-ω6-fed salmon showed significantly poly(I:C)-repressed mRNA levels for *immunoglobulin delta* (*igd*) and *lxra* at 6 hpi and 24 hpi, respectively.

Cluster 2 is comprised of genes significantly induced by poly(I:C) at 24 hpi (Fig. 1a; Supplementary Fig. 2). At 6 hpi, *interferon stimulated gene 15a* (*isg15a*), *signal transducer and activator of transcription 1* paralogues *a* and *b* (*stat1a*, *stat1b*) were significantly poly(I:C)-repressed in salmon fed High-ω6.

Cluster 2 genes showed varying responses to Asal, which were diet-dependent in many cases. *CC chemokine-like 19* (*ccl19*), *interferon gamma* (*ifng*), *interferon alpha* (*ifna*), and *stat1b* were significantly Asal-induced at 24 hpi, and *ccl19* and *interferon regulatory factor 1* paralogues *a* and *b* (*irf1a*, *irf1b*) at 6 hpi in both diet groups. *CC chemokine-like 20* (*ccl20*), *irf1a*, *interferon regulatory factor 7b* (*irf7b*), *stat1a*, *stat1c*, and *toll-like receptor 7* (*tlr7*) only showed significantly Asal-induced mRNA levels in the High-ω3-fed fish at 24 hpi. Other Cluster 2 genes were significantly down-regulated by Asal at 24 hpi in both diet groups [i.e., *toll-like receptor 3* (*tlr3*) and *radical S-adenosyl methionine domain-containing protein 2b* (*rsad2b*)], or only in the group fed High-ω6 [i.e., *irf1b*, *interferon-induced GTP-binding protein b* (*mxb*), *rsad2a*, and *isg15a*]. At 6 hpi, *stat1c* was significantly repressed by Asal in the High-ω3-fed salmon.

In Cluster 3, genes with negative log_2_FCs in the Asal and/or poly(I:C) groups were dominant (Fig. 1a; Supplementary Fig. 3). Some Cluster 3 genes [i.e., *toll-like receptor 5, membrane-bound* (*tlr5m*), *cyclooxygenase-1* (*cox1*), and *arachidonate 5-lipoxygenase* paralogues (*alox5a*, *alox5b*)] showed significantly repressed transcript levels in Asal and poly(I:C)-injected salmon at 24 hpi, regardless of the dietary treatment. Other Cluster 3 genes showed diet-dependent responses to Asal and poly(I:C). *Immunoglobulin mu a* (*igma*) was significantly Asal-repressed in the High-ω6-fed salmon at 24 hpi, whereas *immunoglobulin mu b* (*igmb*) was poly(I:C)-repressed in the High-ω6-fed salmon at 24 hpi. *Prostaglandin-D synthase* (*pgds*) was significantly repressed in the High-ω3-fed salmon 24 h post-Asal injection, in the High-ω6-fed salmon 6 and 24 h post-Asal injection, and in the High-ω6-fed salmon 24 h post-poly(I:C) injection. *Cytochrome P450 family 11 subfamily A member 1* (*cyp11a1*) was significantly down-regulated by Asal and poly(I:C) at 24 hpi only in the High-ω3 group. *Peroxisome proliferator-activated receptor alpha* (*ppara*) was significantly down-regulated by Asal at 24 hpi in the High-ω3-fed salmon and by poly(I:C) at 24 hpi in both diet groups. Asal and poly(I:C) injection significantly induced *cox1* transcript levels at 6 hpi in the High-ω3-fed salmon.

The Principal Component Analysis (PCA) of the log_2_FCs further illustrated the differences in gene expression leading to the classification of the genes into 3 clusters and how these genes effectively separated the treatment [Asal, poly(I:C)] and time (6 and 24 hpi) groups in the multivariate space (Fig. 1c).

### Time-dependent gene expression changes

**Figure 2:**
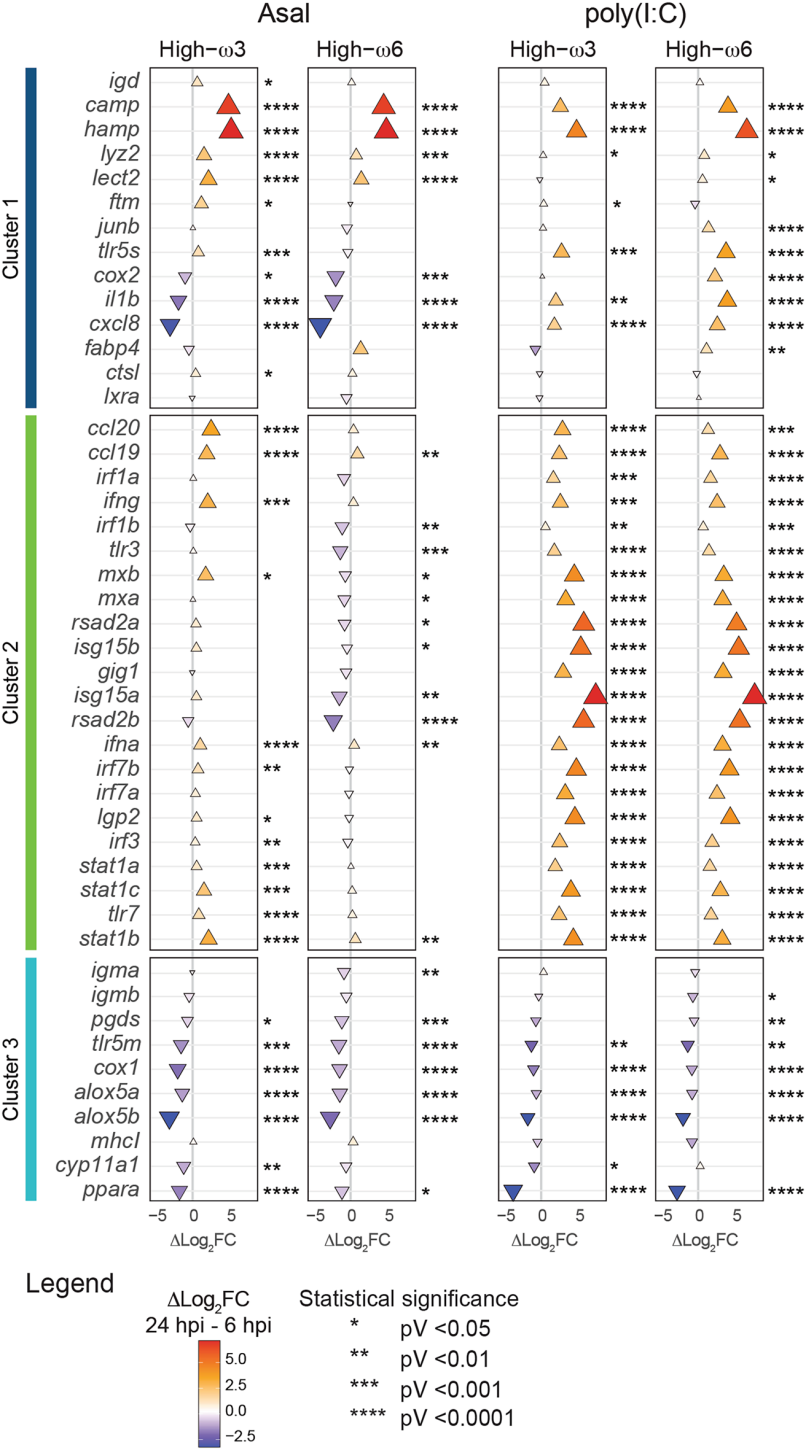
Statistical analyses of the time-dependent changes on the response of 46 immune-relevant genes to formalin-killed *A. salmonicida* (Asal) or polyriboinosinic polyribocytidylic acid [poly(I:C)]. Scatter plot representing the difference between the mean Asal/poly(I:C) log_2_ fold-changes (Δlog_2_FC) at 24 hpi and 6 hpi for each dietary treatment (High-ω3 diet and High-ω6 diet). Upward triangles represent positive Δlog_2_FC (i.e., 24 hpi > 6 hpi); downward triangles represent negative Δlog_2_FC (i.e., 6 hpi > 24 hpi). Asterisks indicate significant differences between time points (n = 7–12). Data normal distribution and homoscedasticity were assessed using the Shapiro–Wilk and Levene’s tests, respectively. Contrast analysis was performed via Student’s t-test (if parametric) or Mann–Whitney U test (if non-parametric or heteroscedastic). An alpha value of 0.05 was applied to all tests.

Asal log_2_FCs significantly increased between 6 and 24 hpi for Cluster 1 genes *camp*, *hamp*, *lyz2*, and *lect2* regardless of the dietary treatment (Fig. 2; Supplementary Data 2; Supplementary Fig. 2). High-ω3-fed salmon showed significantly higher Asal log_2_FCs for *igd*, *ftm*, *tlr5s*, and *ctsl* at 24 hpi than at 6 hpi. Regardless of dietary treatment, Asal log_2_FCs significantly declined between 6 and 24 hpi for *cox2*, *il1b*, and *cxcl8*. No time-dependent differences were observed in the Asal log_2_FCs for *junb* and *fabp4* in both diet groups and *tlr5s* in the High-ω6-fed salmon.

Independent of diet, poly(I:C) log_2_FCs were significantly higher at 24 hpi than at 6 hpi for Cluster 1 genes *camp*, *hamp*, *lyz2*, *tlr5s*, *il1b*, and *cxcl8* (Fig. 2; Supplementary Data 2). In the High-ω3-fed salmon, *ftm* showed significantly increased poly(I:C) log_2_FCs between 6 and 24 hpi. High-ω6-fed salmon showed significantly higher poly(I:C) log_2_FCs for *lect2*, *junb*, *cox2*, and *fabp4* at 24 hpi than at 6 hpi. No time-dependent changes were observed in the poly(I:C) log_2_FCs for *igd*, *ctsl*, and *lxra*.

Within Cluster 2, *ccl19*, *ifna*, *stat1b* showed significantly increased Asal log_2_FCs over time (i.e., 24 hpi > 6 hpi) regardless of the diet (Fig. 2; Supplementary Data 2). High-ω3-fed salmon exhibited significantly higher Asal log_2_FCs for *ccl20*, *ifng*, *mxb*, *irf7b*, *ATP-dependent RNA helicase DHX58* (*lgp2*), *irf3*, *stat1a*, *stat1c*, *tlr7*, and *stat1b* at 24 hpi than at 6 hpi. In the High-ω6-fed salmon, the Asal log_2_FCs for *irf1b*, *tlr3*, *mxb*, *mxa*, *rsad2a*, *isg15b*, *isg15a*, and *rsad2b* decreased significantly between 6 and 24 hpi. The Asal log_2_FCs for *irf1a*, *gig1*, and *irf7a* did not vary with time. In the poly(I:C)-injected salmon, all Cluster 2 genes showed significantly increased log_2_FCs at 24 hpi compared with 6 hpi.

In Cluster 3, *tlr5m*, *cox1*, *alox5a*, *alox5b*, and *ppara* showed significantly reduced Asal/poly(I:C) log_2_FCs at 24 hpi compared with 6 hpi (Fig. 2; Supplementary Data 2) in both diet groups. Except for the poly(I:C)-injected salmon fed the High-ω3 diet, Asal/poly(I:C) log_2_FCs for *pgds* were significantly lower at 24 hpi than at 6 hpi. The Asal/poly(I:C) log_2_FCs for *cyp11a1* decreased significantly over time in the High-ω3 diet salmon. High-ω6-fed salmon showed significantly lower *igma* Asal log_2_FCs and *igmb* poly(I:C) log_2_FCs at 24 hpi than at 6 hpi. No time-dependent changes were observed in the log_2_FCs for *mhcI*.

### Dietary modulation of the immune gene expression responses

**Figure 3:**
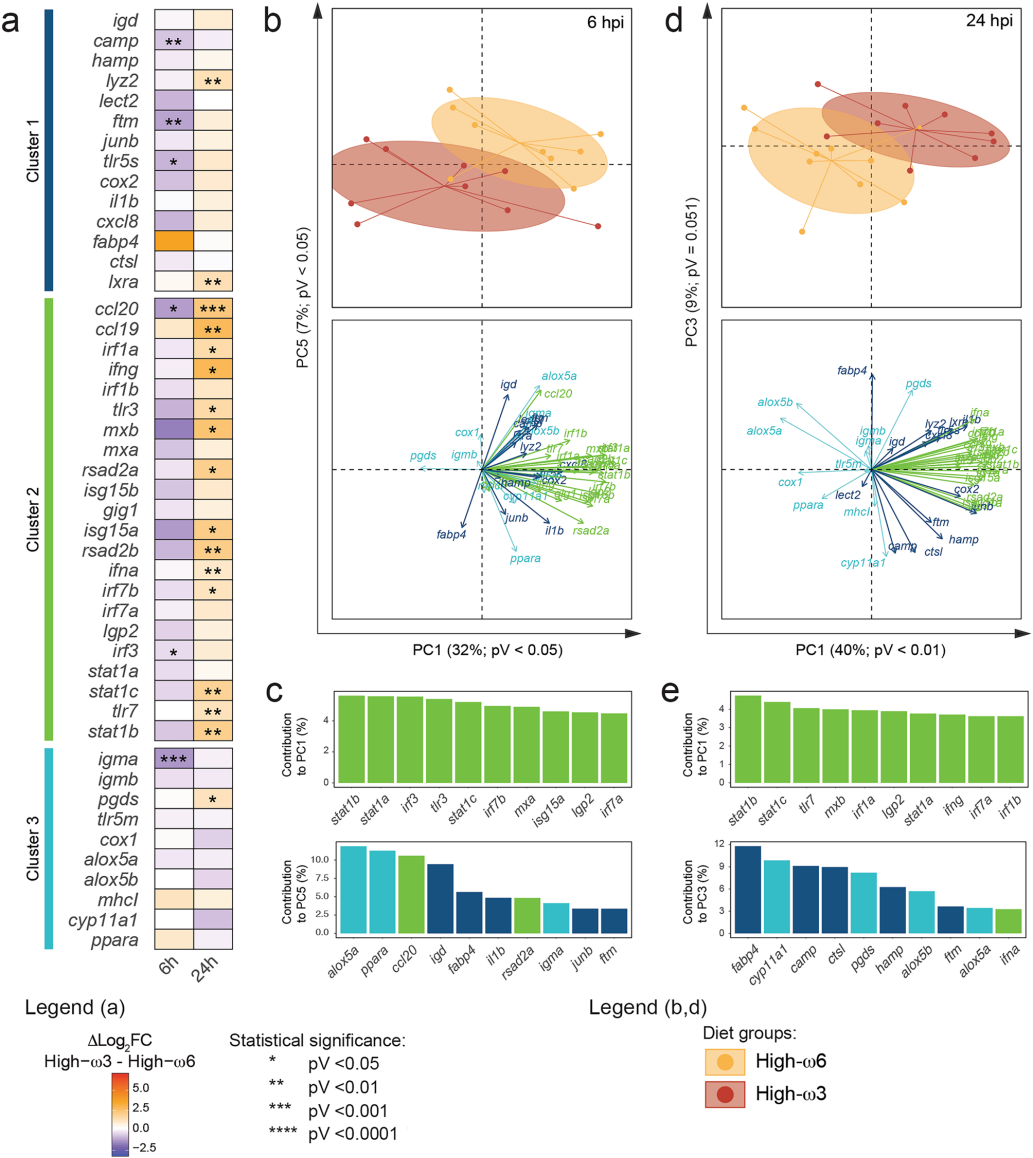
Statistical analyses of the modulatory effects of the experimental diets on the response of 46 immune-relevant genes to formalin-killed *A. salmonicida* (Asal). (**a**) Heatmap representing the difference between the mean log_2_ fold-changes of High-ω3 and High-ω6-fed salmon (Δlog_2_FC) at each time point (6 and 24 hpi). Asterisks indicate significant differences between diet groups (n = 9–10). Data normal distribution and homoscedasticity were assessed using the Shapiro–Wilk and Levene’s tests, respectively. Contrast analysis was performed via Student’s t-test (if parametric) or Mann–Whitney U test (if non-parametric or heteroscedastic). An alpha value of 0.05 was applied to all tests. (**b**) Principal Component Analysis (PCA) plots showing the multivariate distribution of the High-ω3 and High-ω6-fed salmon at 6 h post-Asal injection (top) and the genes’ loading vectors (bottom). PC1 and PC5 were selected because their scores showed the most significant diet-dependent differences. PC scores’ normal distribution and homoscedasticity were assessed using the Shapiro–Wilk and Levene’s tests, respectively. Diet-dependent differences were tested via Student’s t-test (if parametric) or Mann–Whitney U test (if non-parametric or heteroscedastic). An alpha value of 0.05 was applied to all tests. (**c**) Bar plot showing the top 10 genes contributing to the PC1 (top) and PC5 (bottom) variances. The gene’s bars are colored by cluster (see Fig. 1a, b). (**d**) PCA plot for High-ω3 and High-ω6-fed salmons’ multivariate distribution at 24 h post-Asal injection (top) and the genes’ loading vectors (bottom). PC1 and PC3 were selected as the most reflective of the diet effects. PC scores’ normal distribution and homoscedasticity were assessed using the Shapiro–Wilk and Levene’s tests, respectively. Diet-dependent differences were tested using the Student’s t-test (if parametric) or Mann–Whitney U test (if non-parametric or heteroscedastic). An alpha value of 0.05 was applied. (**e**) Bar plot showing the top 10 genes contributing to the PC1 (top) and PC3 (bottom) variances. Bars are colored by cluster.

At 6 hpi (Fig. 3a; Supplementary Data 3; Supplementary Fig. 2), Asal-injected salmon fed High-ω3 tended to have lower log_2_FCs than the High-ω6-fed. These differences were only significant for *camp*, *ftm*, and *tlr5s* in Cluster 1, *ccl20*, and *irf3* in Cluster 2, and *igma* in Cluster 3. At 24 h post-Asal injection, High-ω3-fed salmon showed significantly increased log_2_FCs compared with the High-ω6-fed for *lyz2* and *lxra* in Cluster 1, most Cluster 2 genes (e.g., *ccl20*, *irf1a*, *ifng*, *tlr3*, *mxb*, *rsad2a*, *stat1b*), and *pgds* in Cluster 3.

Components 1 and 5 (PC1 and PC5, respectively) in the 6 hpi/Asal PCA visually and statistically segregated the Asal-injected salmon by diet group (Fig. 3b; Supplementary Data 4). Cluster 2 genes were the top 10 most contributing variables to PC1 (e.g., *stat1b*, *irf3*), whereas the top 10 contributors to PC5 were genes from Clusters 1 (e.g., *igd*, *fabp4*), 2 (e.g., *ccl20*), and 3 (e.g., *alox5a*, *igma*) (Fig. 3c).

In the 24 hpi/Asal PCA (Fig. 3d), PC1 and PC3 visually separated the Asal-injected salmon by dietary treatment, and diet-driven differences were statistically significant for PC1 while close to significant (pV = 0.051) for PC3. The top PC1 contributors were Cluster 2 genes (e.g., *stat1b*, *tlr7*, *mxb*, *irf1a*, *ifng*) (Fig. 3e). The top PC3 contributors were genes from Clusters 1 (e.g., *fabp4*, *camp*, *hamp*), 2 (i.e., *ifna*), and 3 (e.g., *cyp11a1*, *pgds*, *alox5b*).

**Figure 4:**
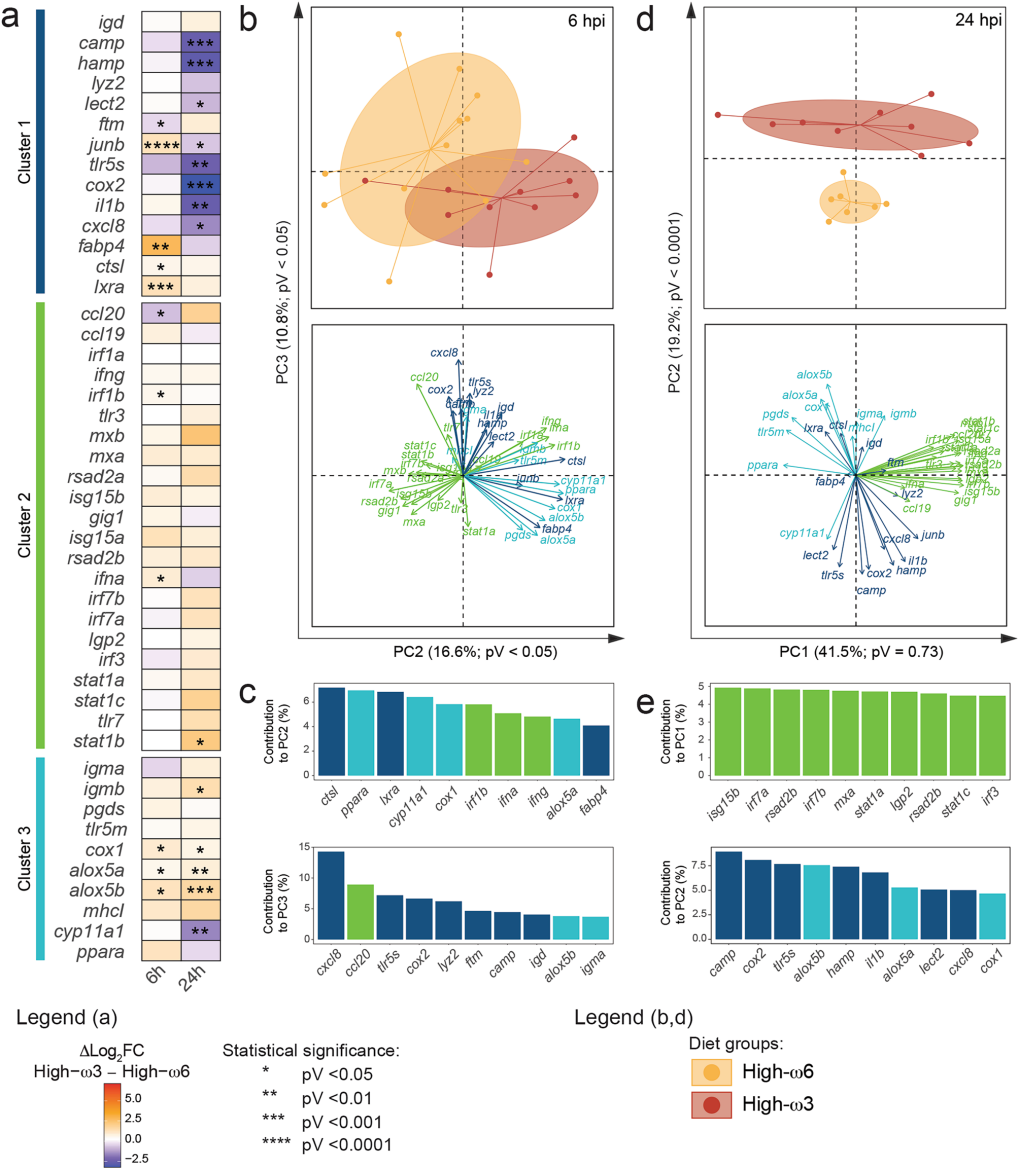
Statistical analyses of the modulatory effects of the experimental diets on the response of 46 immune-relevant genes to polyriboinosinic polyribocytidylic acid [poly(I:C)]. (**a**) Heatmap representing the difference between mean log_2_ fold-changes of High-ω3 and High-ω6-fed salmon (Δlog_2_FC) at each time point (6 and 24 hpi). Asterisks indicate significant differences between diet groups (n = 7–12). Data normal distribution and homoscedasticity were checked using Shapiro–Wilk and Levene’s tests, respectively. Contrast analysis was performed via Student’s t-test (if parametric) or Mann–Whitney U test (if non-parametric or heteroscedastic). An alpha value of 0.05 was applied to all tests. (**b**) Principal Component Analysis (PCA) plots showing the multivariate distribution of the High-ω3 and High-ω6-fed salmon at 6 h post-poly(I:C) injection (top) and the genes’ loading vectors (bottom). PC2 and PC3 were selected since their scores showed the most significant differences between diet groups. PC scores’ normal distribution and homoscedasticity were assessed using the Shapiro–Wilk and Levene’s tests, respectively. Diet-dependent differences were tested using the Student’s t-test (if parametric) or Mann–Whitney U test (if non-parametric or heteroscedastic). An alpha value of 0.05 was applied to all tests. (**c**) Bar plot showing the top 10 genes contributing to the PC2 (top) and PC3 (bottom) variances. The genes’ bars are colored by cluster (see Fig. 1a, b). (**d**) PCA plot for High-ω3 and High-ω6-fed salmon’s multivariate distribution at 24 h post-poly(I:C) injection (top) and the genes’ loading vectors (bottom). PC2 was selected as the only component presenting significant score differences between dietary treatments. PC scores’ normal distribution and homoscedasticity were assessed using the Shapiro–Wilk and Levene’s tests, respectively. Diet-dependent differences were tested using Student’s t-test (if parametric) or Mann–Whitney U test (if non-parametric or heteroscedastic). An alpha value of 0.05 was applied. (**e**) Bar plot showing the top 10 genes contributing to the PC1 (top) and PC2 (bottom) variances. Bars are colored by cluster.

The High-ω3-fed salmon showed a tendency towards higher log_2_FCs for Cluster 3 genes at 6 h post-poly(I:C) injection –significantly higher for *cox1*, *alox5a*, and *alox5b* compared with the High-ω6-fed fish (Fig. 4a). Diet effect was less consistent for Clusters 1 and 2, with genes showing significantly lower (i.e., *ccl20* and *ftm*) or higher (i.e., *irf1b*, *ifna*, *junb*, *fabp4*, *ctsl*, and *lxra*) log_2_FCs in the High-ω3 group.

At 24 hpi (Fig. 4a), High-ω3-fed poly(I:C)-injected salmon had significantly lower log_2_FCs for the Cluster 1 genes *camp*, *hamp*, *lect2*, *junb*, *tlr5s*, *cox2*, *il1b*, and *cxcl8*. In contrast, High-ω3-fed salmon had a tendency towards higher log_2_FCs for Clusters 2 and 3 genes. These differences were significant only for the Cluster 2 gene *stat1b* and Cluster 3 genes *igmb*, *cox1*, *alox5a* and *alox5b*. The exception to this tendency was that High-ω3 significantly reduced *cyp11a1* (Cluster 3) log_2_FCs.

PC1 scores in the 6 hpi/poly(I:C) PCA did not vary with the diet treatment (Supplementary Data 4). In contrast, PC2 and PC3 were able to segregate the poly(I:C)-injected salmon by diet group both visually and statistically (Fig. 4b). The list of top 10 contributors to PC2 showed a balanced composition of genes from all 3 Clusters (e.g., *ctsl*, *ppara*, *ifna*) (Fig. 4c). Among the top 10 contributors to PC3, there were also genes from all Clusters, but Cluster 1 genes were dominant (e.g., *cxcl8*, *tlr5s*).

PC2 was the only component in the 24 hpi/poly(I:C) PCA that showed significantly different scores between diet groups (Fig. 4d). Since all other PCs were noticeably diet-independent, PC1 was selected for the PCA visualization as the one explaining most of the variance. All top 10 contributors to PC1 were Cluster 2 genes (e.g., *isg15b*, *irf7a*), whereas those for PC2 were from Clusters 1 (e.g., *camp*, *cox2*) and 3 (e.g., *alox5b*, *cox1*) (Fig. 4e).

### Head kidney lipid class and fatty acid profiles

The predominant lipid classes in the head kidney samples collected before the immune challenge were triacylglycerol (TAG; 56.1–64.1%), followed by phospholipid (PL; 18.4–20.4%), and sterol (ST; 6.8–11.2%) and acetone-mobile polar lipid (AMPL; 8.2–9.3%) (Table 1; Supplementary Data 5). Feeding the High-ω6 diet resulted in significantly greater ST proportions and ST/PL ratios. No significant differences were found in the other lipid classes.

**Table 1:** Lipid and FA composition of Atlantic salmon head kidney at the end of the 12-week feeding trial.

	High-ω3	High-ω6	pV
Lipid class composition (% of total lipid)			
TAG	64.07 ± 16.16	56.08 ± 15.78	0.132
ST	6.77 ± 4.46	11.24 ± 6.04	< 0.01
AMPL	8.2 ± 7.07	9.32 ± 7.11	0.284
PLγ	18.41 ± 8.84	21.41 ± 10.25	0.385
ST/PL	0.36 ± 0.10	0.58 ± 0.35	< 0.01
FA composition (% of total FAs)			
14:0	1.85 ± 0.25	1.64 ± 0.31	< 0.05
16:0	14.37 ± 1.3	15.27 ± 1.5	0.053
16:1ω7	2.53 ± 0.53	2.27 ± 0.55	0.145
18:0	4.09 ± 0.54	4.61 ± 0.7	< 0.05
18:1ω9 (OA)	21.19 ± 1.88	18.41 ± 4.51	< 0.01
18:1ω7	1.82 ± 0.12	2.16 ± 0.53	< 0.0001
18:2ω6 (LNA)	10.37 ± 1.06	22.99 ± 3.27	< 0.0001
18:3ω3 (ALA)	14.34 ± 2.31	2.37 ± 0.49	< 0.0001
18:4ω3	2.08 ± 0.57	0.84 ± 0.2	< 0.0001
20:1ω9	2.73 ± 0.33	2.54 ± 0.49	0.166
20:2ω6	0.61 ± 0.08	1.48 ± 0.22	< 0.0001
20:3ω6 (DGLA)	0.47 ± 0.12	1.40 ± 0.31	< 0.0001
20:4ω6 (ARA)	0.76 ± 0.33	1.57 ± 0.82	< 0.0001
20:4ω3	1.34 ± 0.26	0.46 ± 0.09	< 0.0001
20:5ω3 (EPA)	3.29 ± 0.86	2.82 ± 0.89	0.134
22:1ω11(13)	2.81 ± 0.53	2.61 ± 0.62	0.282
22:5ω3 (ω3DPA)	0.90 ± 0.11	0.79 ± 0.12	< 0.05
22:6ω3 (DHA)	9.83 ± 3.57	10.79 ± 4.07	0.412
SFA	20.9 ± 1.57	22.3 ± 1.94	< 0.05
MUFA	32.74 ± 3.31	30.33 ± 4.05	< 0.05
PUFA	46.15 ± 2.43	47.18 ± 3.08	0.095
PUFA/SFA	2.22 ± 0.17	2.13 ± 0.19	0.127
Σω3	32.89 ± 2.95	18.32 ± 4.28	< 0.0001
Σω6	12.54 ± 0.75	28.24 ± 2.49	< 0.0001
ω6:ω3	0.39 ± 0.05	1.65 ± 0.51	< 0.0001

Values in cells represent group means ± standard deviation (n = 19–20). Differences between diet groups were assessed via Student’s t-test (if parametric) or Mann–Whitney U test (if not parametric or heteroscedastic). Normal distribution and homoscedasticity were tested using the Shapiro–Wilk and Levene’s tests, respectively. An alpha value of 0.05 was applied to all statistical tests. TAG: triacylglycerol; ST: sterol; AMPL: acetone-mobile polar lipid; PL: phospholipid; OA: oleic acid; LNA: linoleic acid; ALA: α-linolenic acid; DGLA: dihomo-γ-linolenic acid; ARA: arachidonic acid; EPA: eicosapentaenoic acid; ω3DPA: ω3 docosapentaenoic acid; DHA: docosahexaenoic acid; SFA: saturated fatty acids; MUFA: monounsaturated fatty acids; PUFA: polyunsaturated fatty acids.

The High-ω3-fed salmon showed significantly higher proportions of omega-3 FAs in the head kidney, except for 20:5ω3 (EPA) and 22:6ω3 (DHA), which were comparable between the two diet groups. Conversely, the salmon fed High-ω6 had higher omega-6 FA levels, including ARA. The High-ω6-fed salmon had significantly higher SFA and lower MUFA levels. In contrast to the general trend, 14:0 and 18:1ω7 were significantly less and more abundant in the High-ω6-fed salmon, respectively.

## Discussion

Our univariate and multivariate analyses of the transcript expression of 46 immune biomarkers revealed distinctive time- and diet-dependent gene expression responses to Asal and poly(I:C) in the High-ω3-fed and High-ω6-fed salmon.

A suite of previously identified *A. salmonicida*-responsive genes^23–27^ involved in bacterial pathogen recognition (i.e., *tlr5*s)^28^, pro-inflammatory signaling (i.e., *il1b* and *cox2*)^29,30^, neutrophil recruitment (i.e., *lect2*, *cxcl8*)^31^, antimicrobial defense (i.e., *lyz2*, *camp*, *hamp*)^32–34^, immune/stress response regulation (*junb*)^35^, and protein degradation (i.e., *ctsl*)^26^ were grouped in Cluster 1 and were largely induced by Asal at 6 and/or 24 hpi.

Asal induction of *cox2*, *il1b* and *cxcl8* peaked at 6 hpi, whereas that of *camp* and *hamp* intensified between 6 and 24 hpi. Pro-inflammatory mediators and antimicrobial peptides have previously shown similar gene expression changes over time in the head kidney of Atlantic cod IP-injected with formalin-killed *A. salmonicida*^23^ and Atlantic salmon IP-injected with a polyvalent vaccine^36^. While its significance for fish immunity warrants further investigation, this transcript expression pattern appears to follow a logical time course for an immune response i.e., early inflammation signaling (i.e., *cox2* and *il1b*) and neutrophil recruitment (i.e., *cxcl8*) is followed by increased production of antimicrobial peptides (i.e., *camp* and *hamp*).

Genes known to participate in fish antiviral innate responses^15,37,38^ formed Cluster 2. This list included genes encoding viral pathogen recognition receptors (PRRs; i.e., *tlr3*, *tlr7*, and *lgp2*), signal transduction/transcription factors (i.e., *stat1* and *irf* family members), type I and II IFNs (i.e., *ifna* and *ifng*), and effectors [i.e., *mx*, *grass carp reovirus induced gene 1* (*gig1*), *rsad2* and *isg15* paralogues]. These antiviral biomarker genes showed a characteristic induction by poly(I:C) at 24 hpi, in agreement with previous studies in the head kidney and spleen of Atlantic salmon and other teleosts^15,39^. The lymphocyte recruitment-related genes *ccl19* and *ccl20*^40,41^ were also induced at 24 h post-poly(I:C) injection and grouped in Cluster 2.

Genes encoding enzymes involved in the lipid metabolism-inflammation crosstalk (i.e., *pgds*, *cox1*, *alox5a*, *alox5b*, *cyp11a1*, *ppara*)^42–46^, immunoglobulins (i.e., *igma* and *igmb*)^47^, and a PRR (i.e., *tlr5m*)^48^ were grouped in Cluster 3 as characteristically repressed by Asal and poly(I:C) at 24 hpi, in at least one of the diet groups. Decreased transcript levels were previously reported for the eicosanoid synthesis-related genes (i.e., *pgds*, *cox1*, *alox5a*, *alox5b*) in the head kidney of poly(I:C)-injected Atlantic salmon^15^. A gene’s down-regulation can be part of the normal progression of a physiological process. For example, PGDS and ALOX5 participate in the resolution of inflammation through the synthesis of anti-inflammatory eicosanoids (i.e., prostaglandin D_2_ and resolving E2, respectively) in fish and mammals^30,42,43^ –a phase hypothetically not reached by the salmon within the 24-h post-Asal and poly(I:C) injection period based on their pro-inflammatory gene expression signatures (i.e., *il1b*, *cox2*). Besides their lipid metabolism roles, fish and mammalian CYP11A1, and PPARA have also been attributed anti-inflammatory functions^44–46^, so it follows that their activity could be counter-productive during the early stages of the salmon’s response to Asal or poly(I:C).

The repression of *tlr5m* by Asal aligned with the sequential TLR5M/TLR5S signaling model in Atlantic salmon proposed by Muñoz-Flores et al.^48^, where TLR5M’s expression is down-regulated to curb the pro-inflammatory response to flagellin –a potential mechanism to prevent excessive inflammation and collateral tissue damage. The detected lessening of *cox2*, *il1b*, and *cxcl8* induction at 24 h post-Asal injection would also agree with this hypothetical TLR5M-mediated inflammatory response attenuation in Atlantic salmon. Mammalian and fish TLR5 are widely accepted as bacterial flagellin-specific receptors^49^, so it might seem surprising that *tlr5m* and *tlr5s* responded significantly to poly(I:C) at 24 hpi. However, this does not seem to be the case for fish: grass carp (*Ctenopharyngodon idella*) TLR5 has been reported to participate in viral recognition and bind to poly(I:C)^50^.

Some Cluster 1 and 2 genes showed up-regulation by both Asal and poly(I:C). For Cluster 1, both Asal and poly(I:C)-injected salmon showed increased *camp* and *hamp* mRNA levels at 24 hpi. This result could be indicative of an increased presence of antimicrobial peptide-producing cells like neutrophils^51^, agreeing with the up-regulation of inflammation (i.e., *il1b*; *cox2* only in the High-ω6-fed salmon)^29^ and neutrophil recruitment (i.e., *cxcl8*; *lect2* only in the High-ω6-fed salmon)^31^ biomarkers. Except for the poly(I:C)-injected High-ω6-fed salmon, Asal and poly(I:C) induced the Cluster 1 gene *junb* at 6 and 24 hpi, a transcript encoding an activator protein 1 (AP-1) subunit involved in stress responses in mammals^35^ and fish^52,53^. As for Cluster 2, poly(I:C) and Asal injections may have enhanced lymphocyte recruitment at 24 hpi based on the increased *ccl20* and *ccl19* transcript levels^40,41^. Additionally, several Cluster 2 genes related to IFN signaling were induced by Asal. This effect was expected for *ifng* and *stat1* paralogues due to their involvement in pro-inflammatory/cytotoxic immune responses against viral and intracellular bacterial pathogens^38,54^; perhaps not so much for *ifna*, *irf1* paralogues, *irf7b*, and *tlr7*, which are typically classified as antiviral^38,55^. Yet, a growing body of evidence supports the participation of these so-called antiviral genes in teleost antibacterial molecular defense mechanisms^56–60^.

Fighting the invading pathogen becomes a priority for infected organisms –an energetically costly process that requires diverting resources from dispensable processes^61,62^. Some poly(I:C)-inducible genes in Cluster 2 were repressed by Asal, especially at 24 hpi. Among them, there was the gene encoding TLR3, a dsRNA receptor in mammals and fish^37^, which does not seem to play a role in Atlantic salmon immune response to *A. salmonicida*, or in maraena whitefish (*Coregonus maraena*) and rainbow trout (*Oncorhynchus mykiss*) responses to this pathogen^63,64^. However, as reviewed by Rebl et al.^60^, TLR3 might be important in the interactions between other fish and pathogenic bacteria species (e.g., zebrafish infected with *Edwardsiella tarda*). Asal injection also repressed the immune effector-encoding genes *mxb*, *isg15a*, and *rsad2a* in the High-ω6-fed salmon at 24 hpi, and those of *rsad2b* in both diet groups at 24 hpi. Lipopolysaccharides and formalin-killed bacteria (e.g., *Vibrio anguillarum*) have shown varying effects on piscine *mx*, *isg15*, and *rsad2* transcript levels (ranging from up- to down-regulation)^65–67^. Species-specific host–pathogen interactions could explain varied outcomes, alongside dietary influences, as *mxb* and *isg15a* previously exhibited no response to formalin-killed *A. salmonicida* in Atlantic salmon under a different dietary treatment^68^.

Some genes showed opposite directions in their expression at 6 and 24 hpi, in an interestingly diet-dependent manner. In High-ω6-fed salmon, Asal effects on *irf1b* changed from induction at 6 hpi to repression at 24 hpi, whereas poly(I:C) effects on *junb*, *isg15a*, *stat1a*, and *stat1b* shifted from repression (6 hpi) to induction (24 hpi). In High-ω3-fed salmon, *cox1* showed an induction (6 hpi)-to-repression (24 hpi) regulation reversal after Asal and poly(I:C) injection, whereas *stat1c* was repressed at 6 hpi and induced at 24 hpi by Asal. Further research is needed to unravel the immunological significance of these transcript expression shifts for fish infected with viruses and bacteria.

The different ω6:ω3 ratios of the diets tested in the present study significantly changed the relative abundance of immune-relevant FAs in the head kidney lipid composition in Atlantic salmon. As observed in several studies in Atlantic salmon and other teleost species^4,17,18,20^, the FA composition of the head kidney largely reflected that of the diet –i.e., High-ω3 and High-ω6-fed salmon deposited more ω3 (i.e., Σω3) and ω6 (i.e., Σω6) PUFA, respectively.

In terms of eicosanoid-precursor FAs: DGLA (20:3ω6) and ARA (20:4ω6), accumulated with High-ω6 feeding, and ω3DPA (22:5ω3) with High-ω3 feeding, whereas no diet impact was detected on the relative proportion of EPA and DHA. These results agree with our previous findings^17,18^ and suggest promoted endogenous synthesis of these FAs from dietary 18-carbon precursors (i.e., ALA and LNA) in both groups. However, the low dietary ALA (18:3ω3) in the High-ω6-fed salmon suggests increased EPA and DHA retention in that group, as there were no significant differences in tissue proportions. Anti-inflammatory eicosanoids derived from EPA and DGLA interfere with the synthesis of their ARA-derived counterparts and their pro-inflammatory effects^4^. In addition, EPA, ω3DPA and DHA are also precursors for resolvins and protectins, two families of eicosanoids involved in the resolution of inflammation^1,43,69^. Dietary ALA –significantly more abundant in High-ω3-fed salmon– may independently exert anti-inflammatory effects, as observed in humans^70^. Based on the above, the head kidney of the High-ω3-fed salmon would have a more anti-inflammatory ω6 versus ω3 FA profile. In parallel, retaining EPA and DHA and increasing DGLA could have helped the High-ω6-fed salmon balance the potential pro-inflammatory effects from the ARA accumulating in the tissue, as previously proposed^16^.

Despite receiving similar amounts through the diet, High-ω3-fed salmon deposited significantly more oleic acid (OA; 18:1ω9) and the High-ω6-fed more SFAs in their head kidneys. This pattern had been previously observed in the head kidney and liver of Atlantic salmon^18,20^, and might be related to the sparing effect of dietary MUFAs and SFAs on the catabolism of EPA and DHA^71^. Diets rich in *cis* MUFAs and OA have shown similar anti-inflammatory effects to those of FO and DHA in in vivo and in vitro non-human mammalian models^72^. Conversely, SFAs and SFA-rich diets can activate pro-inflammatory signaling pathways in mammals^73^. Therefore, the higher proportion of OA and SFAs may also represent anti-inflammatory and pro-inflammatory traits to the FA profiles of the head kidneys of the High-ω3-fed and High-ω6-fed salmon, respectively.

Dietary lipids are incorporated into cell membranes, thus affecting their structural/chemical properties and function^4,5^. The relative proportion of SFA, MUFA, and PUFA in the PLs, and ST abundance determine cell membrane division into “fluid” (PUFA-rich/ST-poor) and “rigid” (PUFA-poor/ST-rich) domains. Research in mammals revealed that these “rigid” lipid domains (alias lipid rafts) are heavily involved in cell signalling pathways related to immune processes^74^. DHA weakened the TLR22-mediated pro-inflammatory response of yellow croaker (*Larimichthys polyactis*) macrophages to poly(I:C) by disrupting lipid raft formation^75^. Still, the impact of dietary lipids on membrane fluidity and its implications for immunity remain largely unexplored in fish. Here, the detected diet-dependent differences in cell membrane fluidity-relevant lipid parameters (i.e., MUFA and SFA levels and ST/PL ratios) raise the possibility that the diets may have affected immune cell function in the salmon’s head kidneys^4,76^.

High-ω3 restrained the induction of Cluster 1 genes related to inflammation regulation (i.e., *cox2*, *il1b*, and *tlr5s*), neutrophil antimicrobial activity/recruitment (i.e., *camp*, *hamp*, *lect2*, and *cxcl8*), and stress response (i.e., *junb*) at the peak of the pro-inflammatory gene expression response –represented by *cox2* and *il1b*– during the Asal and poly(I:C) challenges, which was observed at 6 hpi for the Asal-injected salmon, and 24 hpi for the poly(I:C)-injected salmon. Concurrently, the High-ω3 diet up-regulated *pgds* in the Asal-injected at 24 hpi and the *alox5* paralogues in the poly(I:C)-injected at 6 and 24 hpi, suggesting promoted synthesis of anti-inflammatory eicosanoids. Furthermore, as highlighted above, the head kidneys of High-ω3-fed salmon showed a theoretically more anti-inflammatory FA profile (i.e., higher ALA, DPA, and OA levels; lower ARA and SFAs levels). In agreement with the present results, lower dietary ω6:ω3 ratios and higher EPA and DHA have previously been seen to attenuate and delay Atlantic salmon’s inflammatory response^8–10^ and repress genes involved in inflammatory/antimicrobial responses (e.g., *tlr5s*^16^, *camp*^9^). In mammals, resolvins derived from ω3 FAs inhibit neutrophil migration^76^.

Interestingly, High-ω3 strengthened the induction of *lyz2* by Asal, the induction of *junb* by poly(I:C), and up-regulated *fabp4* and *lxra* at the low point of *cox2*’s and *il1b*’s response to Asal (24 hpi) and poly(I:C) (6 hpi). *Lyz2* encodes a crucial bacteriolytic enzyme in fish innate immunity^77^, which was previously reported to be constitutively up-regulated in the liver of Atlantic salmon fed higher FO levels^78^. Therefore, High-ω3 might have repressed specific components of Atlantic salmon’s innate immune system [e.g., *cxcl8* at 24 h post-poly(I:C) injection] and promoted others (e.g., *lyz2* at 24 h post-Asal injection). FABP4 and LXRA are involved in the lipid metabolism-inflammation crosstalk in mammalian and fish leukocytes^21,22,79,80^. FABP4 promotes intracellular cholesterol accumulation and pro-inflammatory signaling^79,80^, whereas LXRA promotes cholesterol efflux and inhibits inflammation^21,22^. However, *fabp4* and *lxra* showed inconsistent responses to Asal or poly(I:C) –only salmon fed the High-ω6 showed down-regulated *lxra* transcript levels 24 h post-poly(I:C) injection. While also unresponsive to poly(I:C), *fabp4* was up-regulated in macrophages isolated from head kidneys of Atlantic salmon fed a diet with higher FO content^81^. A higher dietary FO inclusion also increased the transcript levels of *lxra* in the head kidney of healthy and unchallenged Atlantic salmon^20^. All evidence suggests Atlantic salmon *fabp4*’s and *lxra*’s expression is promoted by higher dietary ω3 FA levels –however, their role in the early inflammatory response of Atlantic salmon to viral and bacterial stimulation remains unclear.

Another important finding of the current study is High-ω3 diet up-regulation of Cluster 2 genes belonging to IFN-mediated signaling pathways. This effect was most consistent in the Asal-injected salmon at 24 hpi, with the up-regulation of many IFN-stimulated genes^37^ (e.g., *tlr3*, *mxb*, *rsad2b*), and genes encoding IFNs (i.e., *ifng* and *ifna*) and lymphocyte chemoattractants (i.e., *ccl20* and *ccl19*)^40^. A similar trend –albeit with fewer genes affected– was observed in Cluster 2 genes at 6 and 24 h post-poly(I:C) injection. More specifically, the High-ω3 diet significantly up-regulated *irf1b* and *ifna* at 6 hpi and *stat1b* at 24 hpi. Furthermore, unlike the High-ω6-fed salmon, the High-ω3-fed salmon did not show repression in *isg15a* and *stat1a* at 6 h post-poly(I:C) injection. A previous study on the non-injected salmon fed the High-ω3 diet in this trial revealed up-regulated constitutive hepatic mRNA levels of two *helicase with zinc finger domain 2* paralogues (i.e., *helz2a* and *helz2b*)^19^. Besides its role as a co-activator of PPARA^82^, HELZ2 is also an IFN-stimulated gene involved in mammalian antiviral immune responses^83^. Dietary FO replacement with rapeseed oil was previously found to strengthen *tlr7*, *stat1c*, and *mxb* poly(I:C) induction in the head kidney of Atlantic salmon^15^, and appeared to increase the basal expression of *irf3*, *mxb*, and the antiviral effector *interferon-induced protein with tetratricopeptide repeats 5* (*ifit5*)^84^ in the liver of Atlantic salmon^78^. Rapeseed oil is rich in OA and has an intermediate ALA:LNA ratio (0.6) compared with linseed (4.2) and soy (0.1) oils^6^. Collectively, previous and present findings lead us to hypothesize that vegetable oil-rich diets with high to moderate ALA:LNA ratios (such as 2.3 for High-ω3 here and Katan et al.^19^, and 0.4 for the rapeseed-rich diet tested in Caballero-Solares et al.^15^) might promote the expression of genes involved in Atlantic salmon’s IFN-mediated immune responses.

The dietary manipulation strategy (e.g., same *vs* varying dietary FO levels between diets) and experimental model (e.g., fish species, type of immune challenge) vary substantially among studies, frequently making inter-study comparison challenging. For example, higher dietary inclusion of marine ingredients (fish and krill meals and oils) at the expense of plant ingredients (vegetable protein concentrates and rapeseed oil) was found to be anti-inflammatory and improve the clinical outcome of Atlantic salmon affected by viral inflammatory diseases^8–10^. Despite these findings appearing contradictory to our hypothesis that high vegetable oil diets might enhance Atlantic salmon's antiviral response, it is worth noting that the functional high-marine feeds tested in these previous studies also had lower lipid (energy) content than the control feed. The type and proportions of dietary marine and plant protein sources also differ among studies. Contradictory results among studies investigating the effects of diets/nutrients on fish immune responses (e.g., high-marine diet^8–10^ *vs* high-vegetable oil^15,78^ antiviral properties) are likely indicators of interacting methodological (e.g., total dietary lipid content) and biological (e.g., specific host–pathogen interaction) factors.

In summary, feeding the High-ω3 diet gave rise to Atlantic salmon with anti-inflammatory-biased head kidney FA profiles (e.g., lower ARA content) compared with the High-ω6-fed fish, which agreed with the diminished induction of pro-inflammatory and neutrophil-related biomarkers (e.g., *il1b*, *cox2, cxcl8*, *camp*) in Asal and poly(I:C)-injected salmon. The lipid composition results also indicated differences in cell membrane-related parameters (e.g., ST/PL) between High-ω3-fed and High-ω6-fed salmon that warrant further investigation. Concomitant with its theoretical anti-inflammatory effects, the High-ω3 diet also promoted higher transcript levels of key genes involved in IFN-mediated signaling (e.g., *tlr3*, *ifna*, *irf1b, mxb*, *rsad2b*) in Asal and poly(I:C)-injected salmon. The present and previous research confirm the potential use of vegetable oils to tailor aquafeed lipid composition to stimulate or inhibit specific components of the fish immune system. Diet immunomodulatory properties should necessarily address the specifics of the infection or disease it is intended to prevent or treat –i.e., anti-inflammatory FA profiles might mitigate inflammatory diseases^8–10^ but be deleterious when an early strong inflammatory response is desirable, as in the case of sea lice (*Lepeophtheirus salmonis*) infestation on Atlantic salmon^85^. This study provides new directions for future research on the interaction of fish lipid nutrition and immunity and the exploitation of terrestrial vegetable oils with different ω6: ω3 ratios in developing sustainable low-FO clinical aquaculture feeds.

## Methods

### Feeding trial and experimental diets

The head kidney samples analyzed in the present study were collected in an immune-challenge experiment that followed the feeding trial described in Katan et al.^18^. In the feeding trial, Atlantic salmon smolts from a regional farm (Northern Harvest Sea Farm, Stephenville, NL, Canada) were PIT (Passive Integrated Transponder; Avid Identification Systems, CA, USA)-tagged and acclimated to holding conditions at the Dr. Joe Brown Aquatic Research Building (Ocean Sciences Centre, Memorial University of Newfoundland, NL, Canada) until they were large enough to consume the ~ 5-mm diameter experimental feed pellets. Fish holding conditions consisted of a flow-through filtered seawater system (12 L/min) and a 24-h light photoperiod; water quality was monitored daily and remained constant at 10.1 ± 0.2ºC [mean ± standard deviation (SD)] and 10.1 ± 0.5 mg O_2_/mL. Eighteen days before the beginning of the trial, salmon weighing 203 ± 24 g were randomly distributed—from the 3800-L tank where they were kept since arrival– into twenty 620-L tanks, with 40 fish each. During this acclimation period, the salmon were fed a commercial feed (Winter EP200, Skretting Canada, NB, Canada). Following this, the fish were fed the experimental diets for 12 weeks. Fish growth performance (based on fish weight and fork length) was assessed, and samples for tissue chemical composition (i.e., proximate analysis and fatty acid profiles) and gene expression analyses were collected at the beginning and end of the trial.

In the feeding trial, we tested 5 experimental diets with varying ω6: ω3 ratios –i.e., 1:3 (high ω3 levels), 1:2 (medium ω3 levels), 1:1 (balanced), 2:1 (medium ω6 levels), and 3:1 (high ω6 levels). The different dietary ω6:ω3 ratios were achieved using different proportions of soy and linseed oil while keeping the same FO level for all diets. The feeds were formulated by nutritionists at EWOS Innovation (now Cargill Innovation Center, Dirdal, Norway) to be isonitrogenous and isoenergetic and to meet the nutritional requirements of salmonids^86^. Details on the formulation and chemical composition of the experimental diets can be found in Katan et al.^18^.

### Immune challenge

Katan et al.^18^ investigated the effects of the experimental diets on the transcription of lipid metabolism-related genes and on the lipid composition of liver samples collected in the 12-week feeding trial. For the experiment conducted herein, we selected salmon fed the diets with high omega-3 (High-ω3 diet) and high omega-6 (High-ω6 diet) levels.

As in Caballero-Solares et al.^15^, 20 salmon from these dietary treatments were transferred to new tanks (4 tanks/dietary treatment) and allowed to acclimate for 2 weeks, during which they continued to be fed the same diets. At the end of the acclimation period, salmon weighing 582 ± 98 g were starved for 24 h, and then 2 fish per tank were euthanized by immersing them in a lethal MS-222 bath (400 mg/L of seawater; Syndel Laboratories, Vancouver, BC, Canada), weighed, and dissected for head kidney sample collection. The remaining 18 fish in each tank were lightly anesthetized (50 mg/L MS-222) and subsequently subjected to an intraperitoneal (IP) injection –at a dose of 1 μL/g of fish (wet mass)– with either phosphate-buffered saline (PBS; Ref# 20012027, Gibco/ThermoFisher Scientific, Mississauga, ON, Canada), a solution of polyriboinosinic polyribocytidylic acid [poly(I:C); Ref# P0913, Sigma-Aldrich, Oakville, ON, Canada], or a suspension of formalin-killed *A. salmonicida* (Asal; Furogen Dip, Novartis, Charlottetown, PE, Canada). The poly(I:C) solution was prepared in ice-cold and 0.2 μm-filtered PBS at 2 μg/μL. The Asal suspension was prepared in ice-cold and 0.2 μm-filtered PBS, as described in Hori et al.^24^.

For each diet group, 2 tanks were randomly selected for head kidney sampling at 6 h post-injection (hpi), and the other 2 for 24-hpi head kidney sampling. At the sampling time points, salmon were euthanized by MS-222 overdose (see above), identified using a PIT-tag reader (AVID Power Tracker V, Avid Identification Systems, Calgary, AB, Canada), weighed, and then sampled for head kidney tissue. Head kidney samples were immediately flash-frozen with liquid nitrogen and stored at -80 ºC until processed for RNA extraction.

### Sample selection for RT-qPCR analysis

Similar to our previous study^87^, we utilized samples from individuals highly representative of each tank’s growth performance. Thus, we selected salmon with a weight gain within 1.5*standard deviation below and above their tank mean value. A preliminary RT-qPCR study was conducted to verify whether the Asal and poly(I:C) injections succeeded in eliciting a gene expression immune response. In the preliminary study, *camp* and *cxcl8* transcript levels were measured in the PBS- and Asal-injected salmon. The transcript levels of *isg15a* and *mxb* were measured in the PBS- and poly(I:C)-injected salmon. Based on these analyses, 5 more samples were removed as they were not responsive to Asal or poly(I:C) compared with the PBS controls. One hundred and sixteen samples were finally included in the RT-qPCR experiment –7 to 12 PBS-, Asal-, and poly(I:C)-injected salmon from each diet group and sampling time point.

### RNA preparation and cDNA synthesis

Head kidneys were homogenized in 400 µL of TRIzol Reagent (Invitrogen, Burlington, ON, Canada) using a motorized Kontes RNase-Free Pellet Pestle Grinder (Kimble Chase, Vineland, NJ, USA). An additional 400 µL of TRIzol Reagent (Invitrogen) were added, mixed by pipetting, and the homogenates frozen on dry ice and stored at − 80 °C. Frozen homogenates were further processed by slowly thawing them on ice and then passing them through a QIAshredder (QIAGEN, Mississauga, ON, Canada) spin column following the manufacturer’s instructions. Then, 200 μL of TRIzol (Invitrogen/Life Technologies) were added to each sample to make a total homogenate volume of approximately 1 mL. TRIzol total RNA extractions were then completed following the manufacturer’s instructions.

Total RNA samples (45 µg) were treated with 6.8 Kunitz units of DNaseI (RNase-Free DNase Set, QIAGEN) with the manufacturer’s buffer (1X final concentration) at room temperature for 10 min to degrade any residual genomic DNA. DNase-treated RNA samples were column-purified using the RNeasy Mini Kit (QIAGEN) following the manufacturer’s instructions. RNA integrity was verified by 1% agarose gel electrophoresis, and RNA purity was assessed by A260/280 and A260/230 NanoDrop UV spectrophotometry for both the pre-cleaned and the column-purified RNA samples. Column-purified RNA samples had A260/280 ratios between 2.0 and 2.2 and A260/230 ratios between 1.8 and 2.3.

First-strand cDNA templates for RT-qPCR were synthesized in 20 μL reactions from 1 μg of DNaseI-treated, column-purified total RNA using random primers (250 ng; Invitrogen), dNTPs (0.5 mM final concentration; Invitrogen), and M-MLV reverse transcriptase (200 U; Invitrogen) with the manufacturer’s first strand buffer (1X final concentration) and DTT (10 mM final concentration) at 37 °C for 50 min.

### RT-qPCR

RT-qPCR analyses were conducted following previously published protocols^15,88^ and according MIQE guidelines^89^. PCR amplifications were performed in 13 μL reaction volumes containing 1X Power SYBR Green PCR Master Mix (Applied Biosystems/Life Technologies), 50 nM of both the forward and reverse primers, and the indicated cDNA quantity (see below). The PCR program consisted of 1 cycle of 50 °C for 2 min, 1 cycle of 95 °C for 10 min and 40 cycles of 95 °C for 15 s and 60 °C for 1 min, with fluorescence detection at the end of each 60 °C step and was followed by dissociation curve analysis.

### Selection of genes of interest and normalizer genes

For the RT-qPCR experiment, we selected 46 genes of interest (GOI) involved in fish immunity and that had been previously shown to respond to poly(I:C)^15,81^ and/or bacterial pathogens –including *A. salmonicida*^23–25,27^. The experiment also considered the presence of paralogues in the Atlantic salmon transcriptome as a result of a recent whole-genome duplication in salmonids ~ 80 Mya^90^ and, therefore, used paralogue-specific primers. If classified by putative function, the list of GOIs would comprise 5 pathogen recognition receptors, 7 cytokines, 9 signal transduction/transcription factors, 7 antiviral effectors, 3 antimicrobial peptides, 4 genes involved in adaptive immunity, 5 encoding eicosanoid synthesis enzymes, and 6 genes involved in lipid, protein, and iron metabolism.

For the experimental RT-qPCR study, transcript expression levels of the GOIs were normalized to those of two endogenous control genes. These endogenous controls were selected from six candidate normalizers [*60S ribosomal protein L32* (*rpl32*), *β-actin* (*actb*), *elongation factor 1-alpha 1* (*ef1a1*), *elongation factor 1-alpha 2* (*ef1a2*), *eukaryotic translation initiation factor 3 subunit D* (*eif3d*) and *polyadenylate-binding protein cytoplasmic 1* (*pabpc1*)]. Briefly, the fluorescence threshold cycle (C_T_) values of 48 samples (i.e., 4 individuals for each diet and treatment at each time point) were RT-qPCR-measured in duplicate for each of these transcripts using cDNA representing 4 ng of input total RNA and the 7500 Fast Real Time PCR system (Applied Biosystems), and then analyzed using geNorm^91^. Using this software, *ef1a1* (geNorm M = 0.121) and *rpl32* (geNorm M = 0.154) were determined to be stably expressed in all samples tested and thus selected as normalizers.

### Primer design and performance test

Previously published and newly designed paralogue-specific RT-qPCR primers were used in this study (see Supplementary Data 6). The new primers had been designed following methods described elsewhere^15,88^, and tested for amplification specificity and efficiency using head kidney cDNA from healthy Atlantic salmon (i.e., for *tlr5m*) or from sham-infected and infectious salmon anaemia virus (ISAv)-infected Atlantic salmon (i.e., for *ccl20*, *junb*, *rsad2b*, *cyp11a1*, *ctsl*, and *ftm*). Primer amplification performance was tested using the 7500 Fast Real Time PCR system (Applied Biosystems). Single product amplification and absence of primer-dimer in the no-template control (NTC) were assessed via dissociation curve analysis. Amplicon size was checked electrophoretically on 2% agarose gels and compared with a 1 kb Plus DNA Ladder (Invitrogen). Amplification efficiencies were determined based on a 5-point 1:3 dilution series starting with cDNA representing 10 ng of input total RNA.

### Relative gene expression quantification

RT-qPCR analyses of expression levels of the 46 GOIs in the 116 head kidney samples were performed using the ViiA 7 Real Time PCR system (384-well format) (Applied Biosystems). cDNA representing 4 ng of input total RNA was used as template in the PCR reactions. On each plate, for every sample, the GOIs and endogenous controls were tested in triplicate and a NTC was included. As expression levels of a given gene were measured across multiple plates, a plate linker sample (i.e., a sample that was run on all plates in a given study) was also included to ensure there was no plate-to-plate variability.

The relative quantity (RQ) of each transcript was calculated using the ViiA 7 Software Relative Quantification Study Application (Version 1.2.3; Applied Biosystems). For each GOI, the RQ calculation considered the transcript levels of both *rpl32* and *ef1a1* for the normalization, as well as the amplification efficiency, and set the lowest normalized expression value as the calibrator (i.e., assigned an RQ of 1.0).

### Lipid class and FA analyses

The head kidneys used for lipid class and FA profiling (19–20 salmon for each diet group) were those collected at the end of the 12-week feeding trial (see subsection “Feeding trial and experimental diets” above). These samples were collected, processed, and analysed as described in Hixson et al.^92^. Before use, the tools and glassware used in these procedures were thoroughly lipid-cleaned by rinsing with methanol and chloroform or heating in a muffle furnace. Individual head kidney samples were collected in lipid-clean 15 ml glass test tubes on ice. Sample wet weights were recorded before adding 2 ml of chloroform, filling the tube with nitrogen, closing the tubes with Teflon-lined caps, and sealing with Teflon tape for storage at − 20 °C. Samples were homogenized in a 2:1 ice-cold chloroform:methanol mixture using a Teflon-coated metal rod. Chloroform-extracted water was added to achieve a ratio of chloroform:methanol:water of 8:4:3. The sample was then sonicated (Fisher Scientific FS30, Pittsburgh, PA, USA) for 4 min in an ice bath and centrifuged at 3000 rpm for 2 min at room temperature. The bottom, organic layer was extracted using a double pipetting technique (a 2-ml glass Pasteur pipette inside a 1-ml glass pipette), with chloroform added back to the extraction tube for three repetitions. All organic layers were combined into a lipid-clean vial and concentrated under nitrogen gas flow.

For lipid class composition analysis, an Iatroscan Mark 6 TLC–FID system (Mitsubishi Kagaku Iatron, Inc., Tokyo, Japan) was used with silica-coated Chromarods, with a three-step development method. Lipid extracts were applied to the Chromarods and focused to a narrow band using pure acetone. The first development system was hexane/diethyl ether/formic acid (98.95:1.0:0.05), with the Chromarods being developed for 25 min, removed from the system for 5 min to dry and replaced for 20 min. The second development was for 40 min in hexane/diethyl ether/formic acid (79:20:1, v/v/v). The final development system had two steps: the first was in pure acetone for two 15-min periods, followed by two 10-min periods in a chloroform/methanol/ chloroform-extracted water mixture (5:4:1, v/v/v). Before each solvent system, the Chromarods were dried in a chamber with constant humidity. After each development system, the Chromarods were partially scanned in the Iatroscan, and the data were collected using Peak Simple software (SRI Instruments, version 3.67, Torrance, CA, USA). Calibration of the Chromarods was performed using standards obtained from Sigma Chemicals (Sigma Chemicals, St. Louis, MO, USA).

For FA methyl ester (FAME) analysis, 50 μL of lipid extract were transferred to lipid-clean 15-ml glass vials and concentrated under nitrogen to complete dryness. Subsequently, 1.5 ml of methylene chloride and 3 ml of Hilditch reagent (1.5 sulfuric acid: 98.5 anhydrous methanol) were added, followed by vortexing and a 4-min sonication. Then vials were filled with nitrogen, capped and heated at 100 °C for 1 h. Subsequently, each vial received 0.5 ml of saturated sodium bicarbonate solution and 1.5 ml of hexane. After vortexing, the upper, organic layer was carefully extracted and dried into a separate lipid-clean glass vial. Thereafter, ~ 0.5 ml of hexane was added to each vial, which was also filled with nitrogen, capped, sealed with Teflon tape, and sonicated for 4 min for FA resuspension. All FAMEs were analyzed using an HP 6890 GC-FID system equipped with a 7683 autosampler (Agilent Technologies Canada Inc., Mississauga, Ontario, Canada). Identification of FA peaks was conducted using known standards, including PUFA 1, PUFA 3, BAME, and a Supelco 37 component FAME mixture (Sigma-Aldrich Canada Ltd., Oakville, Ontario, Canada). Finally, chromatograms were integrated using the Varian Galaxie Chromatography Data System (Walnut Creek, CA, USA), and FA data were expressed as the area percentage of FAME.

### Statistical analyses

All data was checked for normal distribution and homoscedasticity using the Shapiro–Wilk and Levene’s tests, respectively, at an alpha value of 0.05. We considered that a given GOI responded significantly to Asal or poly(I:C) when its mean RQ in these groups was significantly different than in the PBS-injected salmon. This contrast analysis and that concerning the differences in lipid composition parameters between diet groups were carried out using Student’s t-test or Mann–Whitney U test at an alpha value of 0.05 for parametric and non-parametric data (with or without equal variances between groups), respectively.

For the analysis of the changes in the response of the GOIs between time points and dietary treatments, the expression fold-change of each Asal/poly(I:C)-injected salmon was calculated by dividing the RQ value of the individual salmon by the mean RQ value of all PBS-injected salmon belonging to the same sampling time point*diet combination. The fold-changes were log_2_-transformed. Log_2_FC differences between 6 and 24 hpi within each diet group and between High-ω3 and High-ω6 within each time point were analyzed via Student’s t-test (if parametric) or Mann–Whitney U test (if not parametric or heteroscedastic) at an alpha value of 0.05.

The Log_2_FC values were also subjected to complete linkage hierarchical clustering and Principal Component Analysis (PCA) to identify common regulatory patterns among the GOIs and visualize how the RT-qPCR data could segregate the salmon by immune-treatment, diet, and time in the multivariate space. The PCA scores of the first 5 principal components were explored for diet effects using Student’s t-test (if parametric) or Mann–Whitney U test (if not parametric or heteroscedastic) at an alpha value of 0.05.

All statistical analyses were conducted using the R environment, more specifically the packages stats (Shapiro–Wilk test, Student’s t-test, and Mann–Whitney U test), car (Levene’s test), and factoextra and ade4 (PCA). Results were plotted using the R packages ggplot2 and ggpubr.

### Ethical approval

All procedures involving salmon handling, treatment, euthanasia, and dissection were performed as per the guidelines of the Canadian Council of Animal Care and approved by the Institutional Animal Care Committee at the Memorial University (Protocol 16-74-MR). This study was performed in compliance with the ARRIVE guidelines (https://arriveguidelines.org).

### Supplementary Information

Supplementary Information.

Supplementary Figures.

## Data Availability

The RT-qPCR data supporting this study’s findings are available at NCBI’s Gene Expression Omnibus (GEO accession number: GSE249779; https://www.ncbi.nlm.nih.gov/geo/query/acc.cgi?acc=GSE249779).
