# Supplementary material for: Vegetable omega-3 and omega-6 fatty acids differentially modulate the antiviral and antibacterial immune responses of Atlantic salmon
Source: Sci Rep. 2024 May 13;14:10947. doi: 10.1038/s41598-024-61144-w (PMC11091188; doi:10.1038/s41598-024-61144-w)
Supplement: Supplementary file 1 — Supplementary Information. [file 41598_2024_61144_MOESM1_ESM.pdf]

Supplementary Data 1 Statistics (t/W), degrees of freedom (df), and p-values (pV) supporting the results shown in Figure 1a concerning the gene expression differences between the Asal/pIC-injected and the PBS-injected salmon.

Treatment		Asal vs PBS																			
Diet		High-ω3										High-ω6									
Time		6 hpi					24 hpi					6 hpi					24 hpi				
Gene of interest	Cluster[1]	Test[2]	t/W	df	pV	Sign.[3]	Test[2]	t/W	df	pV	Sign.[3]	Test[2]	t/W	df	pV	Sign.[3]	Test[2]	t/W	df	pV	Sign.[3]
igd	1	Wilconox	33	-	2.2E-01	NS	Student's	1.7569	14.483	1.0E-01	NS	Wilconox	33	-	3.6E-01	NS	Student's	0.61595	19.999	5.4E-01	NS
camp	1	Student's	11.358	11.633	1.2E-07	****	Wilconox	81	-	4.1E-05	****	Wilconox	90	-	2.2E-05	****	Wilconox	120	-	3.1E-06	****
hamp	1	Wilconox	98	-	4.3E-05	****	Wilconox	81	-	4.1E-05	****	Wilconox	82	-	1.5E-03	**	Wilconox	120	-	3.1E-06	****
lyz2	1	Wilconox	35	-	2.8E-01	NS	Student's	8.026	15.029	8.2E-07	****	Wilconox	35	-	4.5E-01	NS	Student's	2.7126	18.779	1.4E-02	*
lect2	1	Wilconox	30	0	1.4E-01	NS	Student's	6.2523	15.566	1.3E-05	****	Wilconox	34	-	4.0E-01	NS	Student's	5.0537	11.286	3.4E-04	***
ftm	1	Wilconox	46	-	8.0E-01	NS	Student's	1.737	11.03	1.1E-01	NS	Wilconox	73	-	2.2E-02	*	Student's	1.165	11.752	2.7E-01	NS
junb	1	Wilconox	100	-	1.1E-05	****	Wilconox	80	-	8.2E-05	****	Wilconox	85	-	4.1E-04	**	Student's	4.0361	12.77	1.5E-03	**
tlr5s	1	Wilconox	100	-	1.1E-05	****	Wilconox	81	-	4.1E-05	****	Wilconox	90	-	2.2E-05	****	Wilconox	120	-	3.1E-06	****
cox2	1	Wilconox	100	-	1.1E-05	****	Wilconox	81	-	4.1E-05	****	Wilconox	90	-	2.2E-05	****	Wilconox	120	-	3.1E-06	****
il1b	1	Wilconox	100	-	1.1E-05	****	Wilconox	81	-	4.1E-05	****	Wilconox	90	-	2.2E-05	****	Wilconox	120	-	3.1E-06	****
cxcl8	1	Wilconox	100	-	1.1E-05	****	Wilconox	81	-	4.1E-05	****	Wilconox	90	-	2.2E-05	****	Wilconox	120	-	3.1E-06	****
fabp4	1	Wilconox	72	-	1.1E-01	NS	Student's	1.4989	15.967	1.5E-01	NS	Wilconox	45	-	1.0E+00	NS	Student's	0.74911	17.759	4.6E-01	NS
ctsl	1	Student's	1.6184	17.838	1.2E-01	NS	Wilconox	74	-	1.9E-03	**	Student's	2.9978	13.563	9.9E-03	**	Student's	4.5257	17.458	2.8E-04	***
lxr	1	Student's	1.8909	16.085	7.7E-02	NS	Student's	2.053	15.077	5.8E-02	NS	Student's	0.93305	14.577	3.7E-01	NS	Wilconox	35	-	1.1E-01	NS
ccl20	2	Student's	-1.2167	17.296	2.4E-01	NS	Wilconox	81	-	4.1E-05	****	Student's	0.28731	13.252	7.8E-01	NS	Wilconox	83	-	1.4E-01	NS
ccl19	2	Wilconox	88	-	2.9E-03	**	Wilconox	81	-	4.1E-05	****	Wilconox	77	-	7.6E-03	**	Wilconox	119	-	6.2E-06	****
irf1a	2	Wilconox	86	-	5.2E-03	**	Wilconox	91	-	4.1E-05	****	Wilconox	86	-	2.6E-04	***	Wilconox	89	-	5.9E-02	NS
ifng	2	Wilconox	73	-	8.9E-02	NS	Wilconox	81	-	4.1E-05	****	Student's	1.8008	14.528	9.3E-02	NS	Wilconox	107	-	1.1E-03	**
irf1b	2	Student's	2.5351	14.611	2.3E-02	*	Wilconox	52	-	3.4E-01	NS	Student's	3.6046	10.235	4.6E-03	**	Wilconox	28	-	3.6E-02	*
tlr3	2	Wilconox	41	-	5.3E-01	NS	Student's	-2.4139	11.65	3.3E-02	*	Student's	0.23144	12.578	8.2E-01	NS	Student's	-4.7441	17.804	1.7E-04	***
mxb	2	Wilconox	32	-	1.9E-01	NS	Student's	1.0049	15.434	3.3E-01	NS	Student's	-0.85689	16.797	4.0E-01	NS	Wilconox	30	-	4.9E-02	*
mxa	2	Student's	-1.5824	15.722	1.3E-01	NS	Student's	-0.85559	15.164	4.1E-01	NS	Student's	0.47433	15.943	6.4E-01	NS	Student's	-1.8843	17.261	7.6E-02	NS
rsad2a	2	Wilconox	35	-	2.8E-01	NS	Student's	0.071949	15.995	9.4E-01	NS	Student's	-0.48974	16.244	6.3E-01	NS	Student's	-3.1126	19.993	5.5E-03	**
isg15b	2	Student's	0.30956	12.044	7.6E-01	NS	Student's	-0.32118	14.325	7.5E-01	NS	Student's	1.0311	12.752	3.2E-01	NS	Student's	-1.6491	19.284	1.2E-01	NS
gig1	2	Student's	0.092403	13.739	9.3E-01	NS	Student's	-0.46856	12.986	6.5E-01	NS	Student's	0.58102	13.19	5.7E-01	NS	Student's	-1.6869	15.398	1.1E-01	NS
isg15a	2	Wilconox	34	-	2.5E-01	NS	Student's	-0.45764	14.222	6.5E-01	NS	Student's	-0.016015	16.634	9.9E-01	NS	Wilconox	20	-	7.1E-03	**
rsad2b	2	Student's	-1.0974	15.365	2.9E-01	NS	Wilconox	16	-	3.1E-02	*	Wilconox	42	-	8.4E-01	NS	Wilconox	1	-	6.2E-06	****
ifna	2	Wilconox	64	-	3.2E-01	NS	Wilconox	81	-	4.1E-05	****	Student's	2.1198	14.59	5.2E-02	NS	Wilconox	117	-	2.2E-05	****
irf7b	2	Student's	-0.39804	12.949	7.0E-01	NS	Student's	3.4098	14.698	4.0E-03	**	Student's	0.41217	12.391	6.9E-01	NS	Wilconox	65	-	7.7E-01	NS
irf7a	2	Student's	-0.58085	13.103	5.7E-01	NS	Student's	0.22051	15.439	8.3E-01	NS	Student's	-0.52715	14.505	6.1E-01	NS	Student's	-1.6133	19.134	1.2E-01	NS
lgp2	2	Student's	-0.70698	14.991	4.9E-01	NS	Student's	1.5936	15.976	1.3E-01	NS	Student's	0.7387	13.9	4.7E-01	NS	Student's	-0.28439	19.192	7.8E-01	NS
irf3	2	Student's	-1.6301	14.433	1.2E-01	NS	Student's	0.29124	13.597	7.8E-01	NS	Student's	0.18841	13.188	8.5E-01	NS	Student's	-0.91007	19.228	3.7E-01	NS
stat1a	2	Student's	-1.349	16.207	2.0E-01	NS	Student's	2.1578	14.253	4.8E-02	*	Student's	0.54035	12.392	6.0E-01	NS	Student's	1.3728	19.498	1.9E-01	NS
stat1c	2	Student's	-2.3086	13.406	3.7E-02	*	Student's	4.0096	14.922	1.1E-03	**	Student's	-0.41778	14.869	6.8E-01	NS	Student's	0.91875	19.945	3.7E-01	NS
tlr7	2	Student's	-0.97118	16.46	3.5E-01	NS	Wilconox	66	-	2.4E-02	*	Student's	-0.37724	15.911	7.1E-01	NS	Student's	0.481	19.999	6.4E-01	NS
stat1b	2	Student's	-1.3846	12.199	1.9E-01	NS	Student's	6.0998	9.8967	1.2E-04	***	Student's	0.20352	12.615	8.4E-01	NS	Student's	4.5269	18.448	2.5E-04	***
igma	3	Student's	-1.7477	10.223	1.1E-01	NS	Student's	-1.763	14.91	9.8E-02	NS	Student's	2.1492	12.139	5.2E-02	NS	Student's	-2.3455	18.613	3.0E-02	*
igmb	3	Student's	-1.0887	15.023	2.9E-01	NS	Student's	-1.8362	12.293	9.1E-02	NS	Student's	0.028719	16.632	9.8E-01	NS	Student's	-0.98757	19.666	3.4E-01	NS
pgds	3	Wilconox	51	-	9.7E-01	NS	Student's	-4.5489	15.839	3.4E-04	***	Student's	-2.9535	12.002	1.2E-02	*	Student's	-5.072	14.67	1.5E-04	***
tlr5m	3	Student's	0.75682	17.864	4.6E-01	NS	Student's	-8.0439	15.583	6.2E-07	****	Student's	0.86547	12.446	4.0E-01	NS	Student's	-5.7854	17.875	1.8E-05	****
cox1	3	Student's	2.8105	17.242	1.2E-02	*	Student's	-7.4798	12.952	4.7E-06	****	Student's	0.97162	14.529	3.5E-01	NS	Student's	-6.5205	17.852	4.1E-06	****
alox5a	3	Student's	-1.2688	16.607	2.2E-01	NS	Wilconox	0	-	4.1E-05	****	Student's	0.64399	13.827	5.3E-01	NS	Wilconox	0	-	3.1E-06	****
alox5b	3	Student's	-0.43618	17.991	6.7E-01	NS	Wilconox	0	-	4.1E-05	****	Student's	-0.67746	16.941	5.1E-01	NS	Wilconox	0	-	3.1E-06	****
mhcI	3	Student's	0.58438	17.473	5.7E-01	NS	Wilconox	46	-	6.7E-01	NS	Student's	0.78912	11.938	4.5E-01	NS	Wilconox	67	-	6.7E-01	NS
cyp11a1	3	Student's	0.41544	17.973	6.8E-01	NS	Student's	-2.925	13.114	1.2E-02	*	Student's	0.56006	11.43	5.9E-01	NS	Student's	-0.66216	18.494	5.2E-01	NS
ppara	3	Student's	1.4955	16.57	1.5E-01	NS	Student's	-4.1094	11.462	1.6E-03	**	Student's	0.002486	13.143	1.0E+00	NS	Wilconox	39	-	1.8E-01	NS

[1] Cluster the gene was assigned to based on a hierarchical clustering analysis. See Methods section.

[2] Statistical test used for the comparison analysis [Student's t-test (if parametric) or Mann–Whitney U test (if non-parametric or heteroscedastic)]. See Methods section.

[3] Level of significance. NS: not significant; **** for pV < 0.0001; *** for pV < 0.001; ** for pV < 0.01; * for pV < 0.05.

Supplementary Data 1 (Continued)

Treatment		poly(I:C) vs PBS																			
Diet		High-ω3										High-ω6									
Time		6 hpi					24 hpi					6 hpi					24 hpi				
Gene of interest	Cluster[1]	Test[2]	t/W	df	pV	Sign.[3]	Test[2]	t/W	df	pV	Sign.[3]	Test[2]	t/W	df	pV	Sign.[3]	Test[2]	t/W	df	pV	Sign.[3]
igd	1	Student's	1.459	15.482	1.6E-01	NS	Student's	-0.15411	15.995	8.8E-01	NS	Wilconox	95	-	2.1E-02	*	Student's	1.0766	16.015	0.2976	NS
camp	1	Student's	0.72415	17.958	4.8E-01	NS	Wilconox	0	-	4.1E-05	****	Wilconox	63	-	8.7E-01	NS	Wilconox	0	-	3.97E-05	****
hamp	1	Wilconox	57	-	6.3E-01	NS	Wilconox	0	-	4.1E-05	****	Student's	0.35895	19.926	7.2E-01	NS	Wilconox	0	-	3.97E-05	****
lyz2	1	Wilconox	57	-	6.3E-01	NS	Student's	-2.073	9.0505	6.8E-02	NS	Wilconox	64	-	8.2E-01	NS	Student's	-2.1968	8.5222	0.05727	NS
lect2	1	Wilconox	41	-	5.3E-01	NS	Student's	0.47117	15.321	6.4E-01	NS	Wilconox	65	-	7.7E-01	NS	Student's	-2.2174	11.61	0.04736	*
ftm	1	Wilconox	62	-	3.9E-01	NS	Student's	1.3058	11.08	2.2E-01	NS	Wilconox	60	-	1.0E+00	NS	Student's	0.48976	8.755	0.6363	NS
junb	1	Student's	-3.2549	12.651	6.5E-03	**	Wilconox	10	-	5.6E-03	**	Student's	2.3965	18.962	2.7E-02	*	Student's	-7.1907	8.6055	6.46E-05	****
tlr5s	1	Wilconox	57	-	6.3E-01	NS	Wilconox	11	-	7.8E-03	**	Wilconox	59	-	9.7E-01	NS	Wilconox	0	-	3.97E-05	****
cox2	1	Student's	-0.48156	17.734	6.4E-01	NS	Student's	-1.3872	13.67	1.9E-01	NS	Student's	-1.4956	19.215	1.5E-01	NS	Wilconox	0	-	3.97E-05	****
il1b	1	Student's	-1.3039	16.108	2.1E-01	NS	Wilconox	2	-	1.6E-04	***	Student's	-1.4714	19.883	1.6E-01	NS	Wilconox	0	-	3.97E-05	****
cxcl8	1	Wilconox	28	-	3.5E-01	NS	Wilconox	6	-	1.2E-03	**	Student's	-0.17426	19.882	8.6E-01	NS	Wilconox	1	-	7.94E-05	****
fabp4	1	Student's	-1.7576	13.791	1.0E-01	NS	Student's	-0.67327	10.033	5.2E-01	NS	Wilconox	72	-	4.6E-01	NS	Student's	-0.57223	9.9364	0.5799	NS
ctsl	1	Student's	-1.5471	17.641	1.4E-01	NS	Student's	-0.20509	10.211	8.4E-01	NS	Student's	0.59796	19.652	5.6E-01	NS	Student's	2.0936	16.293	0.05228	NS
lxr	1	Student's	-1.8462	14.875	8.5E-02	NS	Student's	0.24424	14.879	8.1E-01	NS	Student's	2.0394	19.524	5.5E-02	NS	Student's	2.4541	13.053	0.02892	*
ccl20	2	Wilconox	76	-	5.2E-02	NS	Wilconox	7	-	1.9E-03	**	Student's	-0.39059	18.473	7.0E-01	NS	Wilconox	6	-	1.19E-03	**
ccl19	2	Student's	-1.2332	15.503	2.4E-01	NS	Wilconox	0	-	4.1E-05	****	Student's	0.54415	17.768	5.9E-01	NS	Wilconox	0	-	3.97E-05	****
irf1a	2	Student's	0.065753	17.191	9.5E-01	NS	Wilconox	0	-	4.1E-05	****	Student's	1.2238	19.338	2.4E-01	NS	Student's	-8.5301	7.3913	4.4E-05	****
ifng	2	Wilconox	44	-	6.8E-01	NS	Wilconox	0	-	4.1E-05	****	Student's	1.4278	14.225	1.7E-01	NS	Wilconox	0	-	3.97E-05	****
irf1b	2	Student's	-0.95026	16.071	3.6E-01	NS	Wilconox	8	-	2.8E-03	**	Student's	1.5418	19.805	1.4E-01	NS	Student's	-3.3459	8.1569	0.009863	**
tlr3	2	Student's	-0.12252	17.73	9.0E-01	NS	Wilconox	3	-	2.9E-04	***	Student's	0.57909	18.953	5.7E-01	NS	Student's	-10.705	11.5	2.51E-07	****
mxb	2	Wilconox	58	-	5.8E-01	NS	Wilconox	0	-	4.1E-05	****	Student's	1.5538	19.997	1.4E-01	NS	Student's	-10.798	6.9452	1.36E-05	****
mxa	2	Wilconox	43	-	6.3E-01	NS	Wilconox	0	-	4.1E-05	****	Wilconox	69	-	5.8E-01	NS	Wilconox	0	-	3.97E-05	****
rsad2a	2	Wilconox	61	-	4.4E-01	NS	Wilconox	0	-	4.1E-05	****	Student's	1.3527	18.094	1.9E-01	NS	Wilconox	0	-	3.97E-05	****
isg15b	2	Student's	-0.73845	14.508	4.7E-01	NS	Wilconox	0	-	4.1E-05	****	Student's	0.88603	19.035	3.9E-01	NS	Wilconox	0	-	3.97E-05	****
gig1	2	Student's	-0.1817	15.725	8.6E-01	NS	Wilconox	0	-	4.1E-05	****	Student's	0.34397	19.718	7.3E-01	NS	Wilconox	0	-	3.97E-05	****
isg15a	2	Student's	-0.39477	17.09	7.0E-01	NS	Wilconox	0	-	4.1E-05	****	Student's	2.5554	15.406	2.2E-02	*	Wilconox	0	-	3.97E-05	****
rsad2b	2	Wilconox	50	-	1.0E+00	NS	Wilconox	0	-	4.1E-05	****	Student's	1.3434	18.831	2.0E-01	NS	Wilconox	0	-	3.97E-05	****
ifna	2	Student's	-1.1859	17.522	2.5E-01	NS	Wilconox	0	-	4.1E-05	****	Student's	0.83555	19.875	4.1E-01	NS	Wilconox	0	-	3.97E-05	****
irf7b	2	Student's	0.77586	12.928	4.5E-01	NS	Wilconox	0	-	4.1E-05	****	Student's	1.8369	19.735	8.1E-02	NS	Wilconox	0	-	3.97E-05	****
irf7a	2	Student's	1.6222	16.546	1.2E-01	NS	Wilconox	0	-	4.1E-05	****	Student's	0.88883	20	3.8E-01	NS	Student's	-13.188	6.6648	5.07E-06	****
lgp2	2	Wilconox	41	-	5.3E-01	NS	Wilconox	0	-	4.1E-05	****	Student's	-0.085423	18.648	9.3E-01	NS	Wilconox	0	-	3.97E-05	****
irf3	2	Student's	1.3619	15.567	1.9E-01	NS	Wilconox	0	-	4.1E-05	****	Student's	1.8983	19.435	7.3E-02	NS	Student's	-8.3721	7.7151	3.89E-05	****
stat1a	2	Student's	1.2406	17.873	2.3E-01	NS	Wilconox	0	-	4.1E-05	****	Student's	3.8094	19.612	1.1E-03	**	Student's	-13.466	9.0773	2.64E-07	****
stat1c	2	Wilconox	66	-	2.5E-01	NS	Wilconox	0	-	4.1E-05	****	Student's	2.079	19.894	5.1E-02	NS	Wilconox	0	-	3.97E-05	****
tlr7	2	Student's	1.978	16.886	6.4E-02	NS	Wilconox	2	-	1.6E-04	***	Student's	1.855	18.142	8.0E-02	NS	Student's	-8.1825	9.9339	1.01E-05	****
stat1b	2	Wilconox	68	-	1.9E-01	NS	Wilconox	0	-	4.1E-05	****	Student's	2.5688	19.984	1.8E-02	*	Wilconox	0	-	3.97E-05	****
igma	3	Student's	0.31778	17.601	7.5E-01	NS	Student's	0.09622	15.174	9.2E-01	NS	Student's	-0.32155	18.073	7.5E-01	NS	Student's	2.1076	11.282	0.0582	NS
igmb	3	Student's	-1.65	10.426	1.3E-01	NS	Student's	0.04568	15.791	9.6E-01	NS	Student's	0.011511	19.889	9.9E-01	NS	Student's	2.8317	15.606	0.01225	*
pgds	3	Wilconox	41	-	5.3E-01	NS	Student's	1.7436	13.19	1.0E-01	NS	Student's	0.86657	17.959	4.0E-01	NS	Student's	3.202	16.384	0.005421	**
tlr5m	3	Student's	0.01076	12.435	9.9E-01	NS	Student's	4.042	12.62	1.5E-03	**	Student's	0.81802	19.701	4.2E-01	NS	Student's	6.3374	16.967	7.5E-06	****
cox1	3	Student's	-2.1411	17.985	4.6E-02	*	Student's	5.7438	10.161	1.8E-04	***	Student's	0.2083	19.754	8.4E-01	NS	Wilconox	84	-	3.97E-05	****
alox5a	3	Student's	-1.2555	15.795	2.3E-01	NS	Student's	3.6327	13.388	2.9E-03	**	Student's	0.89918	17.91	3.8E-01	NS	Student's	9.5367	16.02	5.24E-08	****
alox5b	3	Student's	-1.8125	17.579	8.7E-02	NS	Student's	5.0568	11.342	3.3E-04	***	Student's	1.0524	19.013	3.1E-01	NS	Wilconox	84	-	3.97E-05	****
mhcl	3	Student's	-0.2619	17.726	8.0E-01	NS	Student's	-0.51049	12.517	6.2E-01	NS	Student's	-0.041643	19.988	9.7E-01	NS	Wilconox	44	-	0.9018	NS
cyp11a1	3	Student's	-0.4923	17.829	6.3E-01	NS	Student's	2.7991	11.04	1.7E-02	*	Student's	-0.061709	19.238	9.5E-01	NS	Student's	-0.37973	16.927	0.7089	NS
ppara	3	Student's	-1.2478	17.481	2.3E-01	NS	Wilconox	81	-	4.1E-05	****	Student's	0.45953	17.705	6.5E-01	NS	Wilconox	84	-	3.97E-05	****

[1] Cluster the gene was assigned to based on a hierarchical clustering analysis. See Methods section.

[2] Statistical test used for the comparison analysis [Student's t-test (if parametric) or Mann–Whitney U test (if non-parametric or heteroscedastic)]. See Methods section.

[3] Level of significance. NS: not significant; **** for pV < 0.0001; *** for pV < 0.001; ** for pV < 0.01; * for pV < 0.05.

Supplementary Data 2 Statistics (t/W), degrees of freedom (df), and p-values (pV) supporting the results shown in Figure 2a concerning the log_2-transformed fold-change differences between 6 and 24 hpi.

Treatment		Asal												poly(I:C)											
Diet		High-ω3						High-ω6						High-ω3						High-ω6					
Gene of interest	Cluster[1]	Test[2]	Direction	t/W	df	pV	Sign.[3]	Test[2]	Direction	t/W	df	pV	Sign.[3]	Test[2]	Direction	t/W	df	pV	Sign.[3]	Test[2]	Direction	t/W	df	pV	Sign.[3]
igd	1	Student's	24 hpi > 6 hpi	2.8078	10.948	1.7E-02	*	Wilconox	-	58	-	3.2E-01	NS	Student's	-	1.1732	13.283	2.6E-01	NS	Wilconox	-	60	-	1.4E-01	NS
camp	1	Student's	24 hpi > 6 hpi	24.271	13.362	1.9E-12	****	Student's	24 hpi > 6 hpi	22.241	16.904	5.9E-14	****	Student's	24 hpi > 6 hpi	7.5991	14.675	1.8E-06	****	Student's	24 hpi > 6 hpi	9.2729	13.045	4.2E-07	****
hamp	1	Student's	24 hpi > 6 hpi	9.7086	11.851	5.5E-07	****	Student's	24 hpi > 6 hpi	9.7525	16.192	3.5E-08	****	Student's	24 hpi > 6 hpi	9.4298	15.929	6.4E-08	****	Student's	24 hpi > 6 hpi	15.774	14.604	1.4E-10	****
lyz2	1	Wilconox	24 hpi > 6 hpi	81	-	4.1E-05	****	Student's	24 hpi > 6 hpi	5.061	14.884	1.4E-04	***	Wilconox	24 hpi > 6 hpi	73	-	2.2E-02	*	Student's	24 hpi > 6 hpi	2.57	11.191	2.6E-02	*
lect2	1	Student's	24 hpi > 6 hpi	9.7262	11.402	7.3E-07	****	Student's	24 hpi > 6 hpi	6.802	16.117	4.1E-06	****	Student's	-	-0.27104	16.497	7.9E-01	NS	Student's	24 hpi > 6 hpi	2.5339	16.96	2.1E-02	*
ftm	1	Student's	24 hpi > 6 hpi	2.4073	16	2.9E-02	*	Student's	-	-1.3089	16.491	2.1E-01	NS	Wilconox	24 hpi > 6 hpi	70	-	4.3E-02	*	Student's	-	-0.78896	9.518	4.5E-01	NS
junb	1	Student's	-	-0.81344	15.218	4.3E-01	NS	Student's	-	-1.428	14.016	1.8E-01	NS	Student's	-	1.6331	11.716	1.3E-01	NS	Student's	24 hpi > 6 hpi	10.59	12.147	1.7E-07	****
tlr5s	1	Student's	24 hpi > 6 hpi	4.2451	13.791	8.4E-04	***	Student's	-	-0.17	16.958	8.7E-01	NS	Student's	24 hpi > 6 hpi	4.4571	12.479	7.1E-04	***	Student's	24 hpi > 6 hpi	11.964	15.792	2.5E-09	****
cox2	1	Student's	6 hpi > 24 hpi	-2.7724	11.949	1.7E-02	*	Wilconox	6 hpi > 24 hpi	4	-	2.6E-04	***	Wilconox	-	54	-	5.0E-01	NS	Student's	24 hpi > 6 hpi	6.1746	14.397	2.1E-05	****
il1b	1	Student's	6 hpi > 24 hpi	-11.195	15.506	7.8E-09	****	Student's	6 hpi > 24 hpi	-6.9456	16.284	3.0E-06	****	Student's	24 hpi > 6 hpi	4.1277	12.45	1.3E-03	**	Student's	24 hpi > 6 hpi	11.195	9.897	6.1E-07	****
cxcl8	1	Student's	6 hpi > 24 hpi	-14.952	16	8.0E-11	****	Student's	6 hpi > 24 hpi	-11.449	14.217	1.5E-08	****	Wilconox	24 hpi > 6 hpi	90	-	2.2E-05	****	Student's	24 hpi > 6 hpi	7.326	13.935	3.9E-06	****
fabp4	1	Wilconox	-	28	-	3.0E-01	NS	Wilconox	-	53	-	5.5E-01	NS	Student's	-	-1.2966	15.161	2.1E-01	NS	Student's	24 hpi > 6 hpi	3.0351	14.643	8.5E-03	**
ctsl	1	Student's	24 hpi > 6 hpi	2.7015	13.363	1.8E-02	*	Student's	-	1.7665	16.955	9.5E-02	NS	Student's	-	-1.2032	15.787	2.5E-01	NS	Student's	-	-1.8047	11.973	9.6E-02	NS
lxr	1	Student's	-	0.039527	15.954	9.7E-01	NS	Wilconox	-	22	-	6.5E-02	NS	Student's	-	-1.9427	15.99	7.0E-02	NS	Student's	-	-0.29063	13.883	7.8E-01	NS
ccl20	2	Student's	24 hpi > 6 hpi	7.1976	14.78	3.4E-06	****	Student's	-	0.87439	14.947	4.0E-01	NS	Wilconox	24 hpi > 6 hpi	88	-	8.7E-05	****	Student's	24 hpi > 6 hpi	4.3377	16.739	4.6E-04	***
ccl19	2	Student's	24 hpi > 6 hpi	6.0204	15.678	1.9E-05	****	Student's	24 hpi > 6 hpi	3.3247	16.962	4.0E-03	**	Student's	24 hpi > 6 hpi	8.9405	15.419	1.7E-07	****	Student's	24 hpi > 6 hpi	12.212	14.944	3.6E-09	****
irf1a	2	Student's	-	1.4179	15.894	1.8E-01	NS	Student's	-	-2.0559	16.905	5.6E-02	NS	Student's	24 hpi > 6 hpi	5.429	11.207	1.9E-04	***	Student's	24 hpi > 6 hpi	11.395	15.723	5.3E-09	****
ifng	2	Student's	24 hpi > 6 hpi	4.6079	14.473	3.7E-04	***	Student's	-	1.5915	15.506	1.3E-01	NS	Wilconox	24 hpi > 6 hpi	87	-	1.5E-04	***	Student's	24 hpi > 6 hpi	8.138	11.838	3.4E-06	****
irf1b	2	Student's	-	-0.91736	15.851	3.7E-01	NS	Student's	6 hpi > 24 hpi	-3.3876	15.886	3.8E-03	**	Student's	24 hpi > 6 hpi	3.1568	15.374	6.4E-03	**	Student's	24 hpi > 6 hpi	4.5668	12.715	5.6E-04	***
tlr3	2	Student's	-	-0.46721	12.366	6.5E-01	NS	Student's	6 hpi > 24 hpi	-4.4906	16.345	3.5E-04	***	Student's	24 hpi > 6 hpi	7.2328	13.127	6.3E-06	****	Student's	24 hpi > 6 hpi	9.6898	14.664	9.2E-08	****
mxb	2	Student's	24 hpi > 6 hpi	2.587	15.803	2.0E-02	*	Student's	6 hpi > 24 hpi	-2.4506	15.738	2.6E-02	*	Student's	24 hpi > 6 hpi	9.0256	14.155	3.0E-07	****	Student's	24 hpi > 6 hpi	13.23	15.871	5.5E-10	****
mxa	2	Student's	-	0.01931	16	9.8E-01	NS	Student's	6 hpi > 24 hpi	-2.7557	16.838	1.4E-02	*	Student's	24 hpi > 6 hpi	7.7694	12.862	3.3E-06	****	Student's	24 hpi > 6 hpi	13.485	16.341	2.8E-10	****
rsad2a	2	Student's	-	0.35589	15.474	7.3E-01	NS	Student's	6 hpi > 24 hpi	-2.3196	16.277	3.4E-02	*	Wilconox	24 hpi > 6 hpi	90	-	2.2E-05	****	Student's	24 hpi > 6 hpi	18.028	11.288	1.1E-09	****
isg15b	2	Student's	-	0.29331	13.935	7.7E-01	NS	Student's	6 hpi > 24 hpi	-2.1287	16.902	4.8E-02	*	Student's	24 hpi > 6 hpi	10.841	13.288	5.7E-08	****	Student's	24 hpi > 6 hpi	25.718	16.769	6.5E-15	****
gig1	2	Student's	-	-0.058331	14.651	9.5E-01	NS	Student's	-	-1.4317	15.997	1.7E-01	NS	Student's	24 hpi > 6 hpi	9.1132	14.399	2.3E-07	****	Student's	24 hpi > 6 hpi	19.59	16.901	4.7E-13	****
isg15a	2	Student's	-	0.77744	15.225	4.5E-01	NS	Student's	6 hpi > 24 hpi	-3.5777	16.949	2.3E-03	**	Student's	24 hpi > 6 hpi	15.846	16.978	1.3E-11	****	Student's	24 hpi > 6 hpi	22.054	14.788	1.0E-12	****
rsad2b	2	Student's	-	-1.6011	13.678	1.3E-01	NS	Student's	6 hpi > 24 hpi	-7.5447	16.034	1.2E-06	****	Wilconox	24 hpi > 6 hpi	90	-	2.2E-05	****	Student's	24 hpi > 6 hpi	21.767	9.2976	2.7E-09	****
ifna	2	Student's	24 hpi > 6 hpi	6.1445	15.447	1.6E-05	****	Student's	24 hpi > 6 hpi	3.0964	14.172	7.8E-03	**	Wilconox	24 hpi > 6 hpi	90	-	2.2E-05	****	Student's	24 hpi > 6 hpi	10.889	12.359	1.1E-07	****
irf7b	2	Student's	24 hpi > 6 hpi	4.0751	14.789	1.0E-03	**	Student's	-	0.077158	16.832	9.4E-01	NS	Wilconox	24 hpi > 6 hpi	90	-	2.2E-05	****	Student's	24 hpi > 6 hpi	28.793	16.879	8.7E-16	****
irf7a	2	Student's	-	1.7024	13.951	1.1E-01	NS	Student's	-	-1.1938	16.823	2.5E-01	NS	Wilconox	24 hpi > 6 hpi	90	-	2.2E-05	****	Student's	24 hpi > 6 hpi	20.218	16.849	3.0E-13	****
lgp2	2	Student's	24 hpi > 6 hpi	2.7385	15.735	1.5E-02	*	Student's	-	-0.87616	16.492	3.9E-01	NS	Student's	24 hpi > 6 hpi	15.099	12.201	2.9E-09	****	Student's	24 hpi > 6 hpi	26.503	16.741	4.2E-15	****
irf3	2	Student's	24 hpi > 6 hpi	3.1845	13.693	6.8E-03	**	Student's	-	-1.1133	16.999	2.8E-01	NS	Student's	24 hpi > 6 hpi	8.2742	12.395	2.1E-06	****	Student's	24 hpi > 6 hpi	12.018	13.525	1.3E-08	****
stat1a	2	Student's	24 hpi > 6 hpi	4.3218	14.593	6.4E-04	***	Student's	-	0.80778	15.799	4.3E-01	NS	Student's	24 hpi > 6 hpi	9.1766	10.894	1.9E-06	****	Student's	24 hpi > 6 hpi	19.777	16.051	1.1E-12	****
stat1c	2	Wilconox	24 hpi > 6 hpi	77	-	4.9E-04	***	Student's	-	1.3407	16.96	2.0E-01	NS	Student's	24 hpi > 6 hpi	9.6799	14.215	1.2E-07	****	Student's	24 hpi > 6 hpi	18.292	16.992	1.3E-12	****
tlr7	2	Student's	24 hpi > 6 hpi	5.5998	13.344	7.8E-05	****	Student's	-	0.86703	16.628	4.0E-01	NS	Student's	24 hpi > 6 hpi	9.332	11.66	9.4E-07	****	Student's	24 hpi > 6 hpi	10.928	16.738	4.9E-09	****
stat1b	2	Student's	24 hpi > 6 hpi	6.8235	14.632	6.6E-06	****	Student's	24 hpi > 6 hpi	4.0365	14.052	1.2E-03	**	Student's	24 hpi > 6 hpi	10.18	12.522	2.0E-07	****	Student's	24 hpi > 6 hpi	17.009	16.667	5.8E-12	****
igma	3	Student's	-	-0.5756	10.276	5.8E-01	NS	Student's	6 hpi > 24 hpi	-3.8068	16.11	1.5E-03	**	Student's	-	0.48587	16.513	6.3E-01	NS	Student's	-	-2.1749	9.6918	5.6E-02	NS
igmb	3	Student's	-	-1.9477	12.144	7.5E-02	NS	Student's	-	-1.3291	15.805	2.0E-01	NS	Student's	-	-1.4473	16.237	1.7E-01	NS	Student's	6 hpi > 24 hpi	-2.2784	16.969	3.6E-02	*
pgds	3	Student's	6 hpi > 24 hpi	-2.2158	15.442	4.2E-02	*	Student's	6 hpi > 24 hpi	-5.3827	10.331	2.8E-04	***	Student's	-	-1.806	16.298	8.9E-02	NS	Student's	6 hpi > 24 hpi	-3.5728	8.6402	6.4E-03	**
tlr5m	3	Student's	6 hpi > 24 hpi	-4.6988	15.982	2.4E-04	***	Student's	6 hpi > 24 hpi	-5.2041	16.942	7.2E-05	****	Wilconox	6 hpi > 24 hpi	9	-	2.1E-03	**	Student's	6 hpi > 24 hpi	-4.2413	9.1945	2.1E-03	**
cox1	3	Student's	6 hpi > 24 hpi	-10.44	12.122	2.1E-07	****	Student's	6 hpi > 24 hpi	-7.0429	16.978	2.0E-06	****	Student's	6 hpi > 24 hpi	-9.8739	16.297	2.8E-08	****	Student's	6 hpi > 24 hpi	-8.9208	16.934	8.3E-08	****
alox5a	3	Student's	6 hpi > 24 hpi	-11.443	15.655	5.2E-09	****	Student's	6 hpi > 24 hpi	-12.112	16.96	9.0E-10	****	Student's	6 hpi > 24 hpi	-6.2135	12.477	3.8E-05	****	Student's	6 hpi > 24 hpi	-8.3708	8.1108	2.9E-05	****
alox5b	3	Student's	6 hpi > 24 hpi	-12.329	11.92	3.8E-08	****	Student's	6 hpi > 24 hpi	-19.05	15.373	4.1E-12	****	Student's	6 hpi > 24 hpi	-8.2031	14.344	8.6E-07	****	Student's	6 hpi > 24 hpi	-12.867	10.184	1.3E-07	****
mhcI	3	Wilconox	-	41	-	1.0E+00	NS	Wilconox	-	49	-	7.8E-01	NS	Wilconox	-	46	-	9.7E-01	NS	Wilconox	-	34	-	5.4E-01	NS
cyp11a1	3	Student's	6 hpi > 24 hpi	-3.382	14.708	4.2E-03	**	Student's	-	-0.67477	13.443	5.1E-01	NS	Student's	6 hpi > 24 hpi	-2.818	14.767	1.3E-02	*	Student's	-	0.95762	16.335	3.5E-01	NS
ppara	3	Student's	6 hpi > 24 hpi	-6.0173	15.541	2.0E-05	****	Student's	6 hpi > 24 hpi	-2.2493	16.991	3.8E-02	*	Student's	6 hpi > 24 hpi	-6.0583	11.633	6.5E-05	****	Wilconox	6 hpi > 24 hpi	0	-	4.0E-05	****

[1] Cluster the gene was assigned to based on a hierarchical clustering analysis. See Methods section.

[2] Statistical test used for the comparison analysis [Student's t-test (if parametric) or Mann–Whitney U test (if non-parametric or heteroscedastic)]. See Methods section.

[3] Level of significance. NS: not significant; **** for pV < 0.0001; *** for pV < 0.001; ** for pV < 0.01; * for pV < 0.05.

Supplementary Data 3 Statistics (t/W), degrees of freedom (df), and p-values (pV) supporting the results shown in Figure 3a and 4a concerning the log_2-transformed fold-change differences between the High-ω3 and the High-ω6-fed salmon.

Treatment		Asal												poly(I:C)											
Time		6 hpi						24 hpi						6 hpi						24 hpi					
Gene of interest	Cluster[1]	Test[2]	Direction	t/W	df	pV	Sign.[3]	Test[2]	Direction	t/W	df	pV	Sign.[3]	Test[2]	Direction	t/W	df	pV	Sign.[3]	Test[2]	Direction	t/W	df	pV	Sign.[3]
igd	1	Student's	-	-1.6412	14.64	1.2E-01	NS	Student's	-	1.1389	14.086	2.7E-01	NS	Wilconox	-	67	-	6.7E-01	NS	Student's	-	0.77342	12.41	4.5E-01	NS
camp	1	Student's	High-ω6 > High-ω3	-3.0599	15.259	7.8E-03	**	Student's	-	-0.52725	16.481	6.1E-01	NS	Student's	-	-0.85918	18.72	4.0E-01	NS	Student's	High-ω6 > High-ω3	-4.3431	12.05	9.5E-04	***
hamp	1	Student's	-	-0.29757	13.049	7.7E-01	NS	Student's	-	-0.21994	14.886	8.3E-01	NS	Student's	-	0.14197	19.395	8.9E-01	NS	Student's	High-ω6 > High-ω3	-4.1494	13.977	9.9E-04	***
lyz2	1	Student's	-	-1.0509	11.478	3.1E-01	NS	Student's	High-ω3 > High-ω6	3.3534	14.21	4.6E-03	**	Wilconox	-	54	-	7.2E-01	NS	Student's	-	-0.14184	13.997	8.9E-01	NS
lect2	1	Student's	-	-1.4673	15.992	1.6E-01	NS	Student's	-	0.024958	14.209	9.8E-01	NS	Student's	-	0.045585	19.979	9.6E-01	NS	Student's	High-ω6 > High-ω3	-2.797	13.888	1.4E-02	*
ftm	1	Student's	High-ω6 > High-ω3	-2.9469	15.443	9.8E-03	**	Student's	-	0.60995	16.995	5.5E-01	NS	Wilconox	High-ω6 > High-ω3	28	-	3.6E-02	*	Wilconox	-	39	-	4.7E-01	NS
junb	1	Student's	-	0.13907	13.221	8.9E-01	NS	Student's	-	1.0631	16.222	3.0E-01	NS	Student's	High-ω3 > High-ω6	5.2285	19.435	4.5E-05	****	Student's	High-ω6 > High-ω3	-2.4744	12.909	2.8E-02	*
tlr5s	1	Student's	High-ω6 > High-ω3	-2.1822	14.535	4.6E-02	*	Wilconox	-	57	-	3.6E-01	NS	Student's	-	-1.8501	19.439	8.0E-02	NS	Student's	High-ω6 > High-ω3	-4.2885	11.466	1.2E-03	**
cox2	1	Student's	-	-2.0922	15.98	5.3E-02	NS	Student's	-	0.66207	17	5.2E-01	NS	Wilconox	-	50	-	5.4E-01	NS	Student's	High-ω6 > High-ω3	-5.331	13.932	1.1E-04	***
il1b	1	Wilconox	-	34	-	6.0E-01	NS	Student's	-	1.9899	13.699	6.7E-02	NS	Student's	-	-0.11112	16.885	9.1E-01	NS	Student's	High-ω6 > High-ω3	-3.4417	13.34	4.2E-03	**
cxcl8	1	Student's	-	-2.1596	11.83	5.2E-02	NS	Student's	-	0.76892	16.285	4.5E-01	NS	Student's	-	-1.2269	15.808	2.4E-01	NS	Student's	High-ω6 > High-ω3	-2.3998	13.95	3.1E-02	*
fabp4	1	Student's	-	1.7899	14.878	9.4E-02	NS	Wilconox	-	52	-	6.0E-01	NS	Student's	High-ω3 > High-ω6	3.8269	17.031	1.3E-03	**	Wilconox	-	30	-	9.2E-01	NS
ctsl	1	Student's	-	-1.4922	15.74	1.6E-01	NS	Student's	-	0.005823	14.776	1.0E+00	NS	Student's	High-ω3 > High-ω6	2.1082	17.952	4.9E-02	*	Student's	-	1.9225	13.995	7.5E-02	NS
lxr	1	Wilconox	-	43	-	7.3E-01	NS	Student's	High-ω3 > High-ω6	3.2782	16.915	4.5E-03	**	Student's	High-ω3 > High-ω6	3.8593	19.888	9.9E-04	***	Student's	-	1.7143	13.749	1.1E-01	NS
ccl20	2	Student's	High-ω6 > High-ω3	-2.7399	12.736	1.7E-02	*	Student's	High-ω3 > High-ω6	4.1616	16.694	6.8E-04	***	Student's	High-ω6 > High-ω3	-2.1236	18.052	4.8E-02	*	Student's	-	1.5848	10.746	1.4E-01	NS
ccl19	2	Student's	-	0.44195	15.999	6.6E-01	NS	Student's	High-ω3 > High-ω6	3.065	16.811	7.1E-03	**	Student's	-	1.6593	19.87	1.1E-01	NS	Student's	-	-0.644	13.975	5.3E-01	NS
irf1a	2	Student's	-	-1.042	15.919	3.1E-01	NS	Student's	High-ω3 > High-ω6	2.3618	16.17	3.1E-02	*	Student's	-	0.88282	18.984	3.9E-01	NS	Wilconox	-	31	-	1.0E+00	NS
ifng	2	Student's	-	0.15089	15.919	8.8E-01	NS	Student's	High-ω3 > High-ω6	2.8059	16.89	1.2E-02	*	Student's	-	2.0536	19.648	5.4E-02	NS	Student's	-	0.5701	12.839	5.8E-01	NS
irf1b	2	Student's	-	-1.3065	15.942	2.1E-01	NS	Student's	-	1.7476	14.671	1.0E-01	NS	Student's	High-ω3 > High-ω6	2.1796	18.716	4.2E-02	*	Student's	-	0.9791	13.992	3.4E-01	NS
tlr3	2	Student's	-	-1.427	14.328	1.8E-01	NS	Student's	High-ω3 > High-ω6	2.3157	16.958	3.3E-02	*	Student's	-	0.82247	19.354	4.2E-01	NS	Student's	-	0.81526	9.8505	4.3E-01	NS
mxb	2	Student's	-	-2.1558	11.099	5.4E-02	NS	Student's	High-ω3 > High-ω6	2.7495	14.848	1.5E-02	*	Student's	-	0.56275	19.426	5.8E-01	NS	Wilconox	-	40	-	4.1E-01	NS
mxa	2	Student's	-	-1.6533	14.708	1.2E-01	NS	Student's	-	0.8338	16.321	4.2E-01	NS	Student's	-	0.76483	19.806	4.5E-01	NS	Wilconox	-	37	-	6.1E-01	NS
rsad2a	2	Student's	-	-0.26684	13.013	7.9E-01	NS	Student's	High-ω3 > High-ω6	2.1306	16.766	4.8E-02	*	Wilconox	-	75	-	3.5E-01	NS	Student's	-	0.7361	11.912	4.8E-01	NS
isg15b	2	Student's	-	-1.2641	14.885	2.3E-01	NS	Student's	-	1.1104	16.369	2.8E-01	NS	Student's	-	1.2834	17.386	2.2E-01	NS	Wilconox	-	35	-	7.6E-01	NS
gig1	2	Student's	-	-0.6985	15.915	4.9E-01	NS	Student's	-	0.65968	16.997	5.2E-01	NS	Student's	-	0.32294	17.122	7.5E-01	NS	Wilconox	-	29	-	8.4E-01	NS
isg15a	2	Student's	-	-1.5428	12.418	1.5E-01	NS	Student's	High-ω3 > High-ω6	2.1716	15.509	4.6E-02	*	Student's	-	1.7563	16.651	9.7E-02	NS	Student's	-	0.80775	13.971	4.3E-01	NS
rsad2b	2	Student's	-	-1.5237	13.952	1.5E-01	NS	Student's	High-ω3 > High-ω6	3.2996	16.268	4.4E-03	**	Wilconox	-	75	-	3.5E-01	NS	Wilconox	-	36	-	6.8E-01	NS
ifna	2	Student's	-	-0.94463	11.872	3.6E-01	NS	Student's	High-ω3 > High-ω6	3.0949	16.998	6.6E-03	**	Student's	High-ω3 > High-ω6	2.1518	18.874	4.5E-02	*	Student's	-	-0.96816	13.935	3.5E-01	NS
irf7b	2	Student's	-	-1.0886	15.5	2.9E-01	NS	Student's	High-ω3 > High-ω6	2.8987	16.297	1.0E-02	*	Student's	-	0.41864	17.006	6.8E-01	NS	Wilconox	-	38	-	5.4E-01	NS
irf7a	2	Student's	-	-0.93049	15.553	3.7E-01	NS	Student's	-	1.994	15.441	6.4E-02	NS	Student's	-	-0.46546	19.338	6.5E-01	NS	Wilconox	-	40	-	4.1E-01	NS
lgp2	2	Student's	-	-1.5356	15.484	1.4E-01	NS	Student's	-	2.0218	16.751	5.9E-02	NS	Student's	-	1.0569	19.982	3.0E-01	NS	Wilconox	-	35	-	7.6E-01	NS
irf3	2	Student's	High-ω6 > High-ω3	-2.2963	15.858	3.6E-02	*	Student's	-	1.6261	13.959	1.3E-01	NS	Student's	-	0.13071	18.073	9.0E-01	NS	Student's	-	1.2333	11.764	2.4E-01	NS
stat1a	2	Student's	-	-2.0836	15.964	5.4E-02	NS	Student's	-	1.3957	17	1.8E-01	NS	Student's	-	1.7981	18.448	8.9E-02	NS	Student's	-	1.2879	9.7187	2.3E-01	NS
stat1c	2	Student's	-	-1.642	13.197	1.2E-01	NS	Wilconox	High-ω3 > High-ω6	79	-	4.1E-03	**	Student's	-	0.27576	15.903	7.9E-01	NS	Student's	-	1.8034	9.6665	1.0E-01	NS
tlr7	2	Student's	-	-0.97107	15.937	3.5E-01	NS	Student's	High-ω3 > High-ω6	3.3967	14.996	4.0E-03	**	Student's	-	0.49082	18.741	6.3E-01	NS	Student's	-	1.4548	11.035	1.7E-01	NS
stat1b	2	Student's	-	-1.2656	14.463	2.3E-01	NS	Student's	High-ω3 > High-ω6	3.9812	13.851	1.4E-03	**	Student's	-	0.50801	17.881	6.2E-01	NS	Student's	High-ω3 > High-ω6	2.2572	10.03	4.8E-02	*
igma	3	Student's	High-ω6 > High-ω3	-4.6202	11.597	6.4E-04	***	Student's	-	-0.62978	14.038	5.4E-01	NS	Student's	-	-1.0329	14.682	3.2E-01	NS	Student's	-	1.5477	11.86	1.5E-01	NS
igmb	3	Student's	-	-1.0891	14.499	2.9E-01	NS	Student's	-	-1.033	16.995	3.2E-01	NS	Student's	-	1.5198	16.931	1.5E-01	NS	Student's	High-ω3 > High-ω6	2.2619	13.645	4.1E-02	*
pgds	3	Wilconox	-	48	-	5.5E-01	NS	Student's	High-ω3 > High-ω6	2.8203	16.433	1.2E-02	*	Student's	-	0.91516	14.737	3.7E-01	NS	Student's	-	1.8898	13.343	8.1E-02	NS
tlr5m	3	Student's	-	-0.10414	15.317	9.2E-01	NS	Student's	-	-0.58023	16.088	5.7E-01	NS	Student's	-	1.1003	18.128	2.9E-01	NS	Student's	-	1.2814	13.439	2.2E-01	NS
cox1	3	Student's	-	1.5032	13.168	1.6E-01	NS	Student's	-	-1.123	16.495	2.8E-01	NS	Student's	High-ω3 > High-ω6	2.6927	19.705	1.4E-02	*	Student's	High-ω3 > High-ω6	2.279	13.666	3.9E-02	*
alox5a	3	Student's	-	-1.7177	15.995	1.1E-01	NS	Student's	-	-1.3419	16.763	2.0E-01	NS	Student's	High-ω3 > High-ω6	2.7026	18.521	1.4E-02	*	Student's	High-ω3 > High-ω6	3.2061	13.27	6.7E-03	**
alox5b	3	Student's	-	-0.088172	14.873	9.3E-01	NS	Student's	-	-0.58225	12.95	5.7E-01	NS	Student's	High-ω3 > High-ω6	2.7464	17.675	1.3E-02	*	Student's	High-ω3 > High-ω6	4.2138	13.994	8.7E-04	***
mhcl	3	Wilconox	-	43	-	8.6E-01	NS	Wilconox	-	44	-	9.7E-01	NS	Wilconox	-	68	-	6.3E-01	NS	Wilconox	-	40	-	4.1E-01	NS
cyp11a1	3	Student's	-	0.47684	12.841	6.4E-01	NS	Student's	-	-1.9714	15.542	6.7E-02	NS	Student's	-	0.61848	19.703	5.4E-01	NS	Student's	High-ω6 > High-ω3	-3.8628	12.706	2.0E-03	**
ppara	3	Student's	-	1.9403	14.498	7.2E-02	NS	Student's	-	-0.79722	16.693	4.4E-01	NS	Student's	-	1.8392	19.063	8.2E-02	NS	Student's	-	-0.29625	9.2299	7.7E-01	NS

[1] Cluster the gene was assigned to based on a hierarchical clustering analysis. See Methods section.

[2] Statistical test used for the comparison analysis [Student's t-test (if parametric) or Mann–Whitney U test (if non-parametric or heteroscedastic)]. See Methods section.

[3] Level of significance. NS: not significant; **** for pV < 0.0001; *** for pV < 0.001; ** for pV < 0.01; * for pV < 0.05.

Supplementary Data 4 Statistics (t/W), degrees of freedom (df), and p-values (pV) supporting the results shown in Figure 3b,d and 4b,d concerning the PC score differences between the High-ω3 and the High-ω6-fed salmon.

6 hpi Asal						24 hpi Asal						6 hpi poly(I:C)						24 hpi poly(I:C)					
Component	% Variance	Test[1]	t/W	df	pV	Component	% Variance	Test[1]	t/W	df	pV	Component	% Variance	Test[1]	t/W	df	pV	Component	% Variance	Test[1]	t/W	df	pV
1	31.95	Student's	-2.14	14.64	5.0E-02	1	39.58	Student's	2.935	16.06	9.7E-03	1	27.13	Student's	1.556	19.54	1.4E-01	1	41.45	Student's	0.353	9.39	7.3E-01
2	14.93	Student's	-0.64	13.13	5.3E-01	2	15.03	Student's	0.107	15.49	9.2E-01	2	16.60	Student's	2.533	18.67	2.0E-02	2	19.23	Student's	9.197	13.76	3.0E-07
3	11.21	Student's	-1.28	12.65	2.2E-01	3	9.00	Student's	2.106	16.2	5.1E-02	3	10.84	Student's	-2.17	16.97	4.4E-02	3	8.81	Student's	0.554	11.49	5.9E-01
4	9.13	Student's	1.403	13.87	1.8E-01	4	5.97	Student's	-1.68	16.24	1.1E-01	4	7.85	Student's	-0.02	16.88	9.9E-01	4	6.21	Student's	-0.28	13.81	7.9E-01
5	6.98	Student's	-2.87	16.00	1.1E-02	5	5.34	Wilconox	51	-	6.6E-01	5	6.31	Student's	1.337	14.77	2.0E-01	5	4.86	Student's	-0.05	11.61	9.6E-01

[1] Statistical test used for the comparison analysis [Student’s t-test (if parametric) or Mann–Whitney U test (if non-parametric or heteroscedastic)]. See Methods section.

Supplementary Data 5 Statistics (t/W), degrees of freedom (df), and p-values (pV) supporting the results shown in Table 1 concerning the differences in head kidney lipid composition between the High-ω3 and the High-ω6-fed salmon.

Lipid parameter	Test[1]	Direction	t/W	df	pV	Significance
TAGs	Student's	-	1.5404	35.979	1.3E-01	NS
STs	Wilconox	High-ω3 < High-ω6	90	-	7.5E-03	**
AMPLs	Wilconox	-	143	-	2.8E-01	NS
PLs	Wilconox	-	150	-	3.9E-01	NS
ST/PL	Wilconox	High-ω3 < High-ω6	65	-	4.8E-04	***
14:0	Wilconox	High-ω3 > High-ω6	266	-	3.3E-02	*
16:0	Student's	-	-1.998	36.724	5.3E-02	NS
16:1ω7	Student's	-	1.4896	36.992	1.4E-01	NS
18:0	Student's	High-ω3 < High-ω6	-2.6117	35.678	1.3E-02	*
18:1ω9 (OA)	Wilconox	High-ω3 > High-ω6	286	-	6.3E-03	**
18:1ω7	Wilconox	High-ω3 < High-ω6	20	-	7.9E-08	****
18:2ω6 (LNA)	Student's	High-ω3 < High-ω6	-16.403	23.13	3.1E-14	****
18:3ω3 (ALA)	Student's	High-ω3 > High-ω6	22.102	19.508	2.8E-15	****
18:4ω3	Student's	High-ω3 > High-ω6	9.0594	22.057	6.9E-09	****
20:1ω9	Wilconox	-	240	-	1.7E-01	NS
20:2ω6	Student's	High-ω3 < High-ω6	-16.789	24.402	6.5E-15	****
20:3ω6 (DGLA)	Wilconox	High-ω3 < High-ω6	0	-	2.9E-11	****
20:4ω6 (ARA)	Wilconox	High-ω3 < High-ω6	48	-	2.2E-05	****
20:4ω3	Student's	High-ω3 > High-ω6	14.231	21.783	1.7E-12	****
20:5ω3 (EPA)	Wilconox	-	244	-	1.3E-01	NS
22:1ω11(13)	Wilconox	-	229	-	2.8E-01	NS
22:5ω3 (ω3DPA)	Wilconox	High-ω3 > High-ω6	277	-	1.4E-02	*
22:6ω3 (DHA)	Wilconox	-	160	-	4.1E-01	NS
SFA	Student's	High-ω3 < High-ω6	-2.4775	36.138	1.8E-02	*
MUFA	Student's	High-ω3 > High-ω6	2.0364	36.228	4.9E-02	*
PUFA	Wilconox	-	130	-	9.5E-02	NS
Σω3	Student's	High-ω3 > High-ω6	12.432	33.791	3.7E-14	****
Σω6	Student's	High-ω3 < High-ω6	-26.95	22.571	2.2E-16	****
ω6:ω3	Student's	High-ω3 < High-ω6	-10.994	19.394	8.9E-10	****

[1] Statistical test used for the comparison analysis [Student's t-test (if parametric) or Mann–Whitney U test (if not parametric or heteroscedastic)]. See Methods section.

TAG: triacylglycerol; ST: sterol; AMPL: acetone-mobile polar lipid; PL: phospholipid; OA: oleic acid; LNA: linoleic acid; ALA: α-linolenic acid; DGLA: dihomo-γ-linolenic acid; ARA: arachidonic acid; EPA: eicosapentaenoic acid; ω3DPA: ω3 docosapentaenoic acid; DHA: docosahexaenoic acid; SFA: saturated fatty acids; MUFA: monounsaturated fatty acids; PUFA: polyunsaturated fatty acids.

Supplementary Data 6 Primers used in the RT-qPCR experiment.

Gene name[1]	Gene symbol[1]	GenBank Acc. No.	Forward primer sequence (5' to 3')	Reverse primer sequence (5' to 3')	Efficiency (%)[2]	Amplicon size (bp)	Reference
Viral pathogen recognition receptors							
Toll-like receptor 3	*tlr3*	BK008646	AATATGGCGCTGGTGAAGAG	CGCAAAGGTGAACACTGAGA	102	135	Caballero-Solares et al. 2017. *Fish Shellfish Immunol* 64:24-38. doi: 10.1016/j.fsi.2017.02.040
Toll-like receptor 7	*tlr7*	HF970585	CACCAACACAGAGCTGGAGA	GCCTTGGAAAACTTGCTGAG	86	184	Caballero-Solares et al. 2017. *Fish Shellfish Immunol* 64:24-38. doi: 10.1016/j.fsi.2017.02.040
ATP-dependent RNA helicase DHX58	*lgp2* , alias *dhx58*	BT045378	TCCAAGACCCGTAAAAGCAC	GGTGGAGATCAGGAGGTTGA	93	189	Caballero-Solares et al. 2017. *Fish Shellfish Immunol* 64:24-38. doi: 10.1016/j.fsi.2017.02.040
Bacterial pathogen recognition receptors							
Toll-like receptor 5, secreted	*tlr5s*	AY628755	ATCGCCCTGCAGATTTTATG	GAGCCCTCAGCGAGTTAAAG	96	103	Smith et al. 2018. *Mol Immunol* 95:10-9. doi:10.1016/j.molimm.2018.01.004
Toll-like receptor 5, membrane-bound	*tlr5m*	DY712024	CCTGACCCAGTAGCTTTCCA	TAGTCCCTTTCAGCCACGTC	95	106	This study
Antiviral and pro-inflammatory cytokines							
Interferon alpha	*ifna*	AY216594	TCCGACACCACTACGGTCA	CCTCAACCTCGGCATCAT	100	138	Caballero-Solares et al. 2017. *Fish Shellfish Immunol* 64:24-38. doi: 10.1016/j.fsi.2017.02.040
Interferon gamma	*ifng*	AJ841811	CCGTACACCGATTGAGGACT	GCGGCATTACTCCATCCTAA	103	133	Caballero-Solares et al. 2017. *Fish Shellfish Immunol* 64:24-38. doi: 10.1016/j.fsi.2017.02.040
Interleukin-1 beta	*il1b*	AY617117	GTATCCCATCACCCCATCAC	TTGAGCAGGTCCTTGTCCTT	99	119	Soto-Dávila et al. 2020. *Front Immunol* 10. doi: 10.3389/fimmu.2019.03011
Chemokines							
Interleukin-8	*cxcl8* , alias *il8*	BT046706	GAAAGCAGACGAATTGGTAGAC	GCTGTTGCTCAGAGTTGCAAT	104	99	Soto-Dávila et al. 2020. *Front Immunol* 10. doi: 10.3389/fimmu.2019.03011
Leukocyte cell-derived chemotaxin-2	*lect2*	BT059281	CAGATGGGGACAAGGACACT	GCCTTCTTCGGGTCTGTGTA	99	150	Smith et al. 2018. *Mol Immunol* 95:10-9. doi:10.1016/j.molimm.2018.01.004
CC chemokine-like 19	*ccl19*	BT058161	CTGCTTGACAACGACCGATA	GTTGTTCTTGGTGGCAGGAG	95	151	Caballero-Solares et al. 2017. *Fish Shellfish Immunol* 64:24-38. doi: 10.1016/j.fsi.2017.02.040
CC chemokine-like 20	*ccl20*	EG862627	TATCCAGGCATGGTCAGGTT	ATGTTTTGACCCCTGACCTTTA	95	100	This study
Signal transduction and gene transcription regulation							
Signal transducer and activator of transcription 1a	*stat1a*	BT045567	GACTGGGAAAATGTGGCTGT	CATGTGAACAGGGTCCTCCT	100	180	Caballero-Solares et al. 2017. *Fish Shellfish Immunol* 64:24-38. doi: 10.1016/j.fsi.2017.02.040
Signal transducer and activator of transcription 1b	*stat1b*	BT048927	GTTCAGGATGCAGAGCATGA	CCATCCCATTCACCTCTTGT	99	109	Caballero-Solares et al. 2017. *Fish Shellfish Immunol* 64:24-38. doi: 10.1016/j.fsi.2017.02.040
Signal transducer and activator of transcription 1c	*stat1c*	DW551983	GGTCCACACAAATCAACGTG	CTTTGCAGGGCCTTCTTCTT	103	154	Caballero-Solares et al. 2017. *Fish Shellfish Immunol* 64:24-38. doi: 10.1016/j.fsi.2017.02.040
Interferon regulatory factor 1a	*irf1a*	BT048538	GCAATGAAGTAGGCACAGCA	CGCAGCTCTATTTCCGTTTC	100	100	Caballero-Solares et al. 2017. *Fish Shellfish Immunol* 64:24-38. doi: 10.1016/j.fsi.2017.02.040
Interferon regulatory factor 1b	*irf1b*	FN806856	CGCGTTTACAAAATGCTGAA	GGTTGCGCTGTAGTCCATTT	105	123	Caballero-Solares et al. 2017. *Fish Shellfish Immunol* 64:24-38. doi: 10.1016/j.fsi.2017.02.040
Interferon regulatory factor 3	*irf3*	NM_001172282	ACAAACAGCTGGGAACCAAC	ATTGGATATTGCCGTTGCTC	102	108	Caballero-Solares et al. 2017. *Fish Shellfish Immunol* 64:24-38. doi: 10.1016/j.fsi.2017.02.040
Interferon regulatory factor 7a	*irf7a*	BT045216	CCAGTGCCACCAGTCCTAAT	GGTGATCTCCAAGTCCCAGA	95	105	Caballero-Solares et al. 2017. *Fish Shellfish Immunol* 64:24-38. doi: 10.1016/j.fsi.2017.02.040
Interferon regulatory factor 7b	*irf7b*	FJ517644	GTCAGTGGTAAAATCAACACGC	CACCATCATGAAACGCTTGGT	91	99	Caballero-Solares et al. 2017. *Fish Shellfish Immunol* 64:24-38. doi: 10.1016/j.fsi.2017.02.040
Transcription factor JunB	*junb*	BT044843	CCGAGAACTATCGGAACCAA	GGATGATCAATCGCTCCAGT	101	129	This study
Antiviral effectors							
Interferon-induced GTP-binding protein a	*mxa*	U66475	CTGAAAAGCGGAGTTCGTCT	CTCCCTCGATCCTCTGGTTA	91	112	Caballero-Solares et al. 2017. *Fish Shellfish Immunol* 64:24-38. doi: 10.1016/j.fsi.2017.02.040
Interferon-induced GTP-binding protein b	*mxb*	BT044881	ACGCACCACTCTGGAGAAAT	CTTCCATTTCCCGAACTCTG	94	184	Caballero-Solares et al. 2017. *Fish Shellfish Immunol* 64:24-38. doi: 10.1016/j.fsi.2017.02.040
Interferon stimulated gene 15a	*isg15a*	BT049918	AAAGTGGCCACAACAAAGCAG	ATAGGAGCGGGCTCCGTAATC	90	140	Caballero-Solares et al. 2017. *Fish Shellfish Immunol* 64:24-38. doi: 10.1016/j.fsi.2017.02.040
Interferon stimulated gene 15b	*isg15b*	DY731374	CTTTGGAGTGGCCATGGAAAG	GGATATGAGTGGGTTCTGTAAT	99	146	Caballero-Solares et al. 2017. *Fish Shellfish Immunol* 64:24-38. doi: 10.1016/j.fsi.2017.02.040
Radical S-adenosyl methionine domain-containing protein 2a	*rsad2a* , alias *viperina*	BT047610	GTACCGCAGATGCACAACAC	GCACTGTCGGGTAAAATGGT	104	135	This study
Radical S-adenosyl methionine domain-containing protein 2b	*rsad2b* , alias *viperinb*	DY728694	TTCCTGGCATGGATAGGTGT	CTTGGAGTTGTCGCTGGTTT	102	113	Zanuzzo et al. 2020. *Front Immunol* 11. doi:10.3389/fimmu.2020.01009
Grass carp reovirus induced gene 1	*gig1*	BT044028	GTTCTGGGTTTGGTCGTCAC	CTGTTCTGGAAGGGATGGAA	88	151	Caballero-Solares et al. 2017. *Fish Shellfish Immunol* 64:24-38. doi: 10.1016/j.fsi.2017.02.040
Antimicrobial peptides							
Cathelicidin antimicrobial peptide	*camp*	AY360357	AGACTGGCAACACCCTCAAC	TTGCCTCTTCTTGTCCGAAT	103	112	Eslamloo et al. 2020. *Fish Shellfish Immunol* 98:937–49. doi:10.1016/j.fsi.2019.11.057
Hepcidin	*hamp*	BT125319	ATGAATCTGCCGATGCATTTC	AATGGCTTTAGTGCTGGCAG	96	134	Eslamloo et al. 2020. *Fish Shellfish Immunol* 98:937–49. doi:10.1016/j.fsi.2019.11.057
Lysozyme C II	*lyz2*	BT047934	ATACGGAATGGATGGCTACG	AGATGCCATAGTCGGTGGAG	91	125	Xue et al. 2020. *Mar Biotechnol* , *22* , 263-284. doi:10.1007/s10126-020-09950-x
Adaptive immunity							
Major histocompatibility complex class I	*mhcI*	AF504022	CATGAAGATGTGGAGCATGG	AGACCCGTGACTTGAACCAC	95	131	Caballero-Solares et al. 2017. *Fish Shellfish Immunol* 64:24-38. doi: 10.1016/j.fsi.2017.02.040
Immunoglobulin delta	*igd*	AF141606	ACCCTAGGAGTTCAACTGGAAA	AAACCTGCAACAGGAAAATGTA	106	138	Caballero-Solares et al. 2018. *BMC Genomics* , 19, 1-26. doi:10.1186/s12864-018-5188-6
Immunoglobulin mu a	*igma*	BT058702	AGCATTCACTTGCGTGTTTG	CGGGGTGATCTTAATGACTACT	104	115	Caballero-Solares et al. 2018. *BMC Genomics* , 19, 1-26. doi:10.1186/s12864-018-5188-6
Immunoglobulin mu b	*igmb*	BT059185	GAAGTTTCATTCACTTGCGTGT	GCGGGATGATGTTAATGACC	99	121	Caballero-Solares et al. 2018. *BMC Genomics* , 19, 1-26. doi:10.1186/s12864-018-5188-6
Eicosanoid metabolism							
Cyclooxygenase-1	*cox1* , alias *ptgs1*	BT045745	CTCATGAGGGTGGTCCTCAC	AGGCACAGGGGGTAGGATAC	102	135	Caballero-Solares et al. 2017. *Fish Shellfish Immunol* 64:24-38. doi:10.1016/j.fsi.2017.02.040
Cyclooxygenase-2	*cox2* , alias *ptgs2*	AY848944	ACCTTTGTGCGAAACGCTAT	GAGTAGGCCTCCCAGCTCTT	109	113	Caballero-Solares et al. 2017. *Fish Shellfish Immunol* 64:24-38. doi:10.1016/j.fsi.2017.02.040
Prostaglandin-D synthase	*pgds* , alias *lipocalin-type*	BT048787	GGTGCTCAACAAGCTCTACA	GCAGGAAAGCGATGTTGTCA	93	114	Caballero-Solares et al. 2017. *Fish Shellfish Immunol* 64:24-38. doi:10.1016/j.fsi.2017.02.040
Arachidonate 5-lipoxygenase a	*alox5a* , alias *5loxa*	NM_001139832	CTGCTCACCATGCTGCTGTC	GTGTGGGAGGAGGCTTCC	96	93	Caballero-Solares et al. 2017. *Fish Shellfish Immunol* 64:24-38. doi:10.1016/j.fsi.2017.02.040
Arachidonate 5-lipoxygenase b	*alox5b* , alias *5loxb*	CX354498	ACTGCTGTGGGTTTCCCAAG	GACAGCAGCGTGATGTGCAG	98	98	Caballero-Solares et al. 2017. *Fish Shellfish Immunol* 64:24-38. doi:10.1016/j.fsi.2017.02.040
Lipid, protein, and iron metabolism							
Cytochrome P450 Family 11 Subfamily A Member 1	*cyp11a1*	CA063876	CTTCAGGAGCTTGGGCTTC	GGTACTGTGCACCTCCACCT	97	142	This study
Oxysterols receptor LXR-alpha	*lxra*	GE770391	CTCGCCTGTGTTCCTGTTTT	GAAACGCAAGACCTTCTGCT	102	103	Katan et al. 2020. *Front Mol Biosci* 14. doi:10.3389/fmolb.2020.602587
Fatty acid-binding protein, adipocyte	*fabp4*	NM_001141203	GACTTGGGACGGCAAGACTA	CAGCAGACTGGAATCACACC	98	128	Eslamloo et al. 2017. *BMC genomics* , 18, 1-28. doi:10.1186/s12864-017-4099-2
Peroxisome proliferator-activated receptor alpha	*ppara*	NM_001123560	GAACGTGACCCACATCGAG	TGACCAGCTGACGGAGGT	90	137	This study
Cathepsin L1	*ctsl*	BT057383	GTTCAGGAAGACTGGCAAGC	GGCGTTGTCCTGGATGTACT	100	130	This study
Ferritin middle subunit	*ftm*	BT058199	GCAGAATAGTCGGAGGAACTTT	CTTCGCAATCGTGGTGATAG	96	109	This study
Normalizers							
Elongation factor 1 alpha 1	*ef1a1*	AF321836	TGGCACTTTCACTGCTCAAG	CAACAATAGCAGCGTCTCCA	98	197	Smith et al. 2018. *Mol Immunol* 95:10-9. doi:10.1016/j.molimm.2018.01.004
60S ribosomal protein 32	*rpl32*	BT043656	AGGCGGTTTAAGGGTCAGAT	TCGAGCTCCTTGATGTTGTG	91	119	Xue et al. 2015. *Comp Biochem Physiol Part D* 14:1-15. doi:10.1016/j.cbd.2015.01.005

[1] Name and symbol of the qPCR-analyzed genes interest. Putative human orthologue symbols were assigned based on HUGO Gene Nomenclature Committee (HGNC; https://www.genenames.org/) and/or GeneCards (https://www.genecards.org/).
[2] Amplification efficiencies were calculated using a 5-point 1:3 dilution series starting with cDNA representing 10 ng of input total RNA.
