# Supplementary figures and images for: Vegetable omega-3 and omega-6 fatty acids differentially modulate the antiviral and antibacterial immune responses of Atlantic salmon
Source: Sci Rep. 2024 May 13;14:10947. doi: 10.1038/s41598-024-61144-w (PMC11091188; doi:10.1038/s41598-024-61144-w)

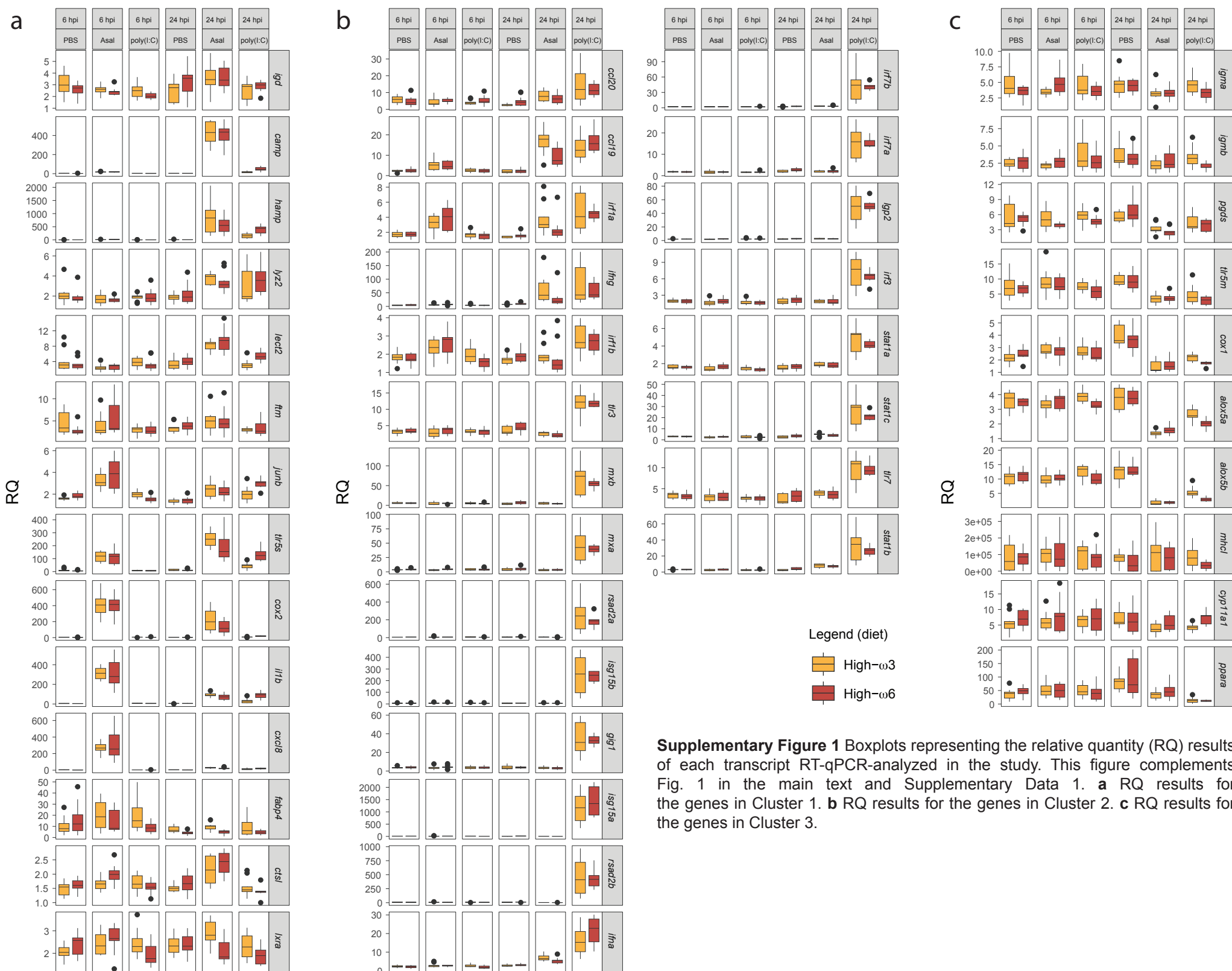

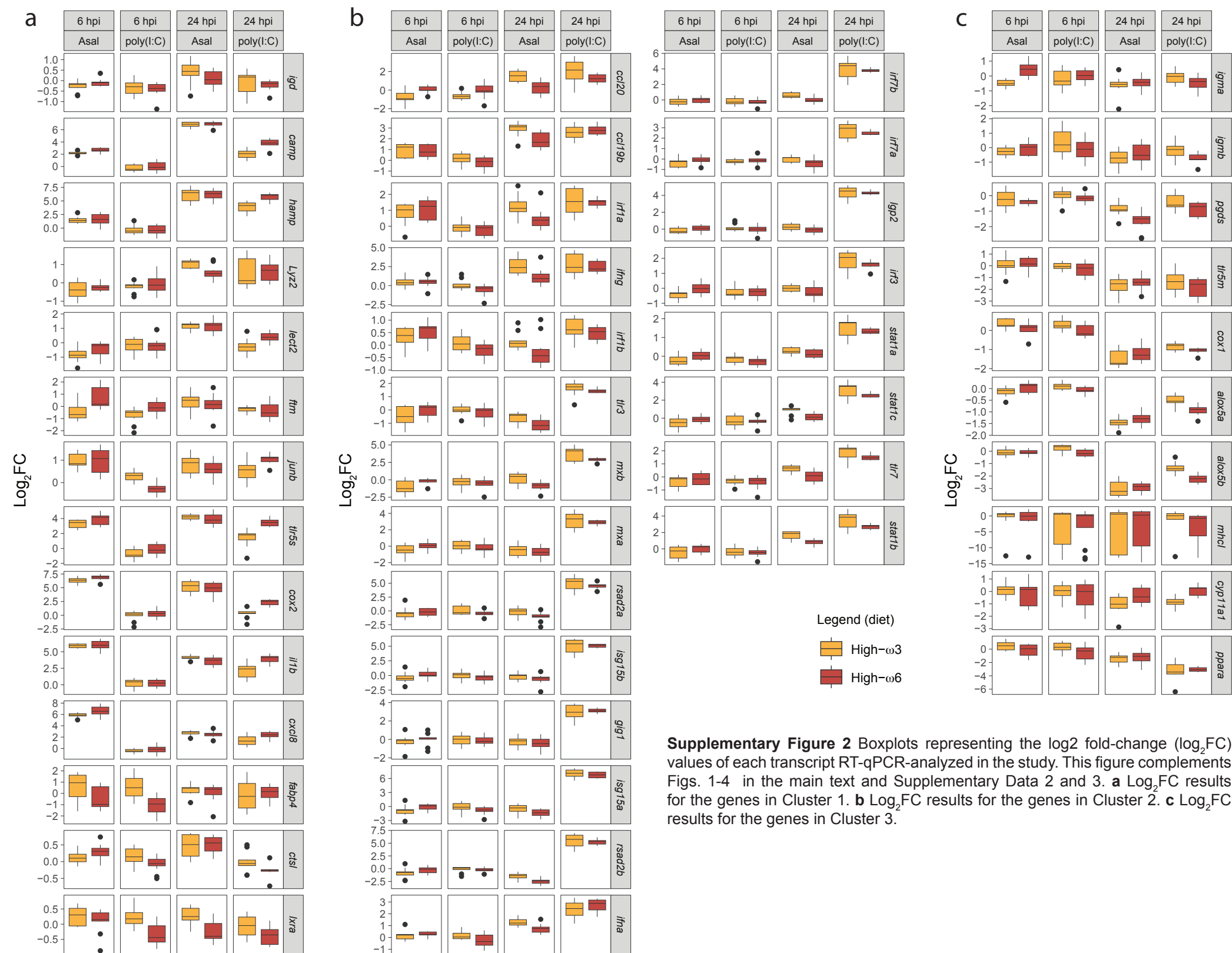

Supplement: Supplementary file 2 — Supplementary Figures. [file 41598_2024_61144_MOESM2_ESM.pdf]
